# Summarising data and factors associated with COVID-19 related conspiracy theories in the first year of the pandemic: a systematic review and narrative synthesis

**DOI:** 10.1186/s40359-022-00959-6

**Published:** 2022-11-01

**Authors:** Konstantinos Tsamakis, Dimitrios Tsiptsios, Brendon Stubbs, Ruimin Ma, Eugenia Romano, Christoph Mueller, Ayesha Ahmad, Andreas S. Triantafyllis, George Tsitsas, Elena Dragioti

**Affiliations:** 1 Institute of Psychiatry, Psychology and Neuroscience (IoPPN), King’s College London, London, UK; 2 Second Department of Psychiatry, Attikon University General Hospital, National and Kapodistrian University of Athens, Athens, Greece; 3 Institute of Medical and Biomedical Education, St George’s University of London, London, UK; 4 Neurology Department, Democritus University of Thrace, Alexandroupolis, Greece; 5 South London and Maudsley NHS Foundation Trust, Denmark Hill, London, UK; 6 Department of Cardiology, Asklepeion General Hospital Athens, Athens, Greece; 7 Counselling Centre, Harokopio University, Athens, Greece; 8 Department of Health, Medicine and Caring Sciences, Pain and Rehabilitation Centre, Linköping University, Linköping, Sweden

**Keywords:** Conspiracy theories, Beliefs, COVID-19, Pandemic, First year, Public health, Misinformation, Infodemic

## Abstract

Conspiracy theories can have particularly harmful effects by negatively shaping health-related behaviours. A significant number of COVID-19 specific conspiracy theories emerged in the immediate aftermath of the pandemic outbreak. The aim of this study was to systematically review the literature on conspiracy theories related to COVID-19 during the first year of the pandemic (2020), to identify their prevalence, their determinants and their public health consequences. A comprehensive literature search was carried out in PubMed and PsycINFO to detect all studies examining any conspiracy theory related to COVID-19 between January 1st 2020, and January 10th 2021. Forty-three studies were included with a total of 61,809 participants. Between 0.4 and 82.7% of participants agreed with at least one conspiracy belief. Certain sociodemographic factors (young age, female gender, being non-white, lower socioeconomic status), psychological aspects (pessimism, blaming others, anger) and other qualities (political conservatism, religiosity, mistrust in science and using social media as source of information) were associated with increased acceptance of conspiracy theories. Conspiracy beliefs led to harmful health-related behaviours and posed a serious public health threat. Large-scale collaborations between governments and healthcare organizations are needed to curb the spread of conspiracy theories and their adverse consequences.

**Supplementary Information:**

The online version contains supplementary material available at 10.1186/s40359-022-00959-6.

## Background

Conspiracy theories constitute “a conviction that a group of actors meets in secret agreement with the purpose of attaining some malevolent goal’’ [1] and/or provide explanations for important events and circumstances involving such secret conspiracies [2]. Although holding onto conspiracy beliefs has been considered almost pathological (‘the paranoid style’ in the 1960s) [3] and has been linked to paranoia and cynicism [4], there are large portions of the ‘normal’ population who actually do believe in conspiracy theories [1, 4]. In early 2020, as the COVID-19 pandemic loomed, several conspiracy theorists promoted misinformation on social media forums, including statements such as 1) ‘SARS-CoV-2 is a bio-weapon generated in Wuhan, China that was accidentally or deliberately released worldwide by the Chinese’, 2) ‘5G telecommunication network activates the virus’, 3) ‘the pandemic is a hoax perpetrated by a global cabal’, and 4) ‘Bill Gates deliberately created SARS-CoV-2 to make profits by selling vaccines, or to launch a broad vaccination program to facilitate a global surveillance regime’ [5, 6]. At the same time, several anti-vaccination rhetors rejected the efficacy and safety of anti-SARS-CoV-2 vaccines, instead promoting unproven therapies such as homoeopathic arsenic-based products, colloidal silver solutions, prophylactic vitamin megadoses, garlic, hot pepper and lemon to limit the effects of the pandemic [7].

The most dangerous implication of this widespread misinformation is that COVID-19-related conspiracy theories negatively influence not only preventive behaviours, but also societal attitudes towards anti-COVID-19 vaccination regimes, leading to further global spread of this deadly virus [6, 8]. Despite this, the literature on COVID-19 conspiracy theories has largely not been systematically reviewed to date, with the exception of a review of anti-vaccination conspiracy theories [9], a very recent systematic review that included the period when vaccines had already become available [10] and a meta-analysis which focused more on the role of conspiracy beliefs for COVID-19 health responses[11]. Additionally, systematically reviewed evidence on pre-COVID-19 conspiracy theories remains scarce [12]. Therefore, we conducted a systematic review of all available literature investigating COVID-19-related conspiracy theories to map their prevalence (i.e., how widespread they were) in the first year of the pandemic (2020), when new information about COVID-19 was continuously emerging and evolving [13], and before systematic vaccination of the population began. The second overarching goal of this review was to identify relevant factors and population characteristics that influence their existence. A third aim was to summarise existing evidence on the potential consequences of these conspiracy theories.

## Materials and methods

We followed the Preferred Reporting Items for Systematic Reviews and Meta-analyses (PRISMA checklist) as a guidance for this study (see Additional file 1: Checklist 1). A protocol has not been registered; however, our study methods were designed and formulated a priori.

### Literature search strategy

A comprehensive literature search was conducted by two investigators (ED, KT) independently in PubMed and PsycINFO to trace all relevant studies published between January 1^st^, 2020, and January 10^th^, 2021. We included any quantitative and qualitative study that examined any conspiracy theory (as measured by any scale) related to COVID-19 outbreak. The search strategy was “(conspiracy theories OR conspiracy theory OR conspiracy beliefs OR science denial OR scepticism) AND (COVID-19 OR SARS-CoV-2 OR coronavirus OR corona virus)” (see Additional file 1: Box1). We did not apply restrictions regarding language, country, ethnicity, or any other characteristic during the search process. The retrieved articles were also hand screened for other potentially suitable articles. Any disagreements regarding the screening, or selection process were solved by a third investigator (GT) until a consensus was reached.

### Eligibility criteria

We included peer reviewed observational studies (quantitative and qualitative) and randomized studies, if the latter existed, published in the English language (although the search was done without language restrictions as mentioned above) that examined associations between COVID-19 and conspiracy theories. Studies analysing social media feed, such as tweets related to COVID-19 conspiracy theories were also eligible for inclusion.

Studies were excluded if they: 1) provided insufficient or inadequate data for descriptive and quantitative synthesis, 2) were letters to the editors, commentaries, viewpoints, and 3) were irrelevant to the SARS-CoV-2.

### Data extraction

Data extraction was performed independently by two investigators from the team (RA and ER) using a predefined data form created in Excel. The two same investigators also rated the quality of the included studies using the AHRQ (Agency for Healthcare Research and Quality) checklist, since the majority of the studies was cross-sectional (Additional file 1: Table 1). The AHRQ checklist consists of 11 items, with classifications of ‘yes’, ‘no’, or “unclear’. The studies are classified as “high quality” (8–11 items with a ‘yes’ response); moderate quality (4–7 items with a ‘yes’ response); and “low quality” (0–3 items with a ‘yes’ response) [14]. For the qualitative studies, we used the CASP Qualitative Research Checklist, adapted from Horntvedt et al. [15] with moderate and high methodological quality defined as meeting 6–8 and 9–10 of the CASP respectively (See Additional file 1: Table 2).

We recorded author, year, country, study design, sampling method, sample size, mean age of participants, % female, ethnicity (if possible), type of conspiracy, measurement of conspiracy theories, measurement of other variables, mean and standard deviation per conspiracy instrument (if possible), % believers in conspiracy theories of the study sample. We also obtained data on the main findings and relevant socio-demographic (e.g., gender, income, political views) and psychological factors, as well as consequences and repercussions associated with conspiracy theories. Possible discrepancies during data extraction were solved with discussion with a third investigator (KT).

### Data analysis

No statistical analysis or meta-analysis were performed due to the high heterogeneity of the studies. Thus, the data were only descriptively analysed. In particular, we used a narrative synthesis approach, which refers to an approach to systematically review and synthesize results from multiple studies, relying mainly on the use of words and text to summarise and explain the results of the synthesis [16].

## Results

### Database searches

**Fig. 1:**
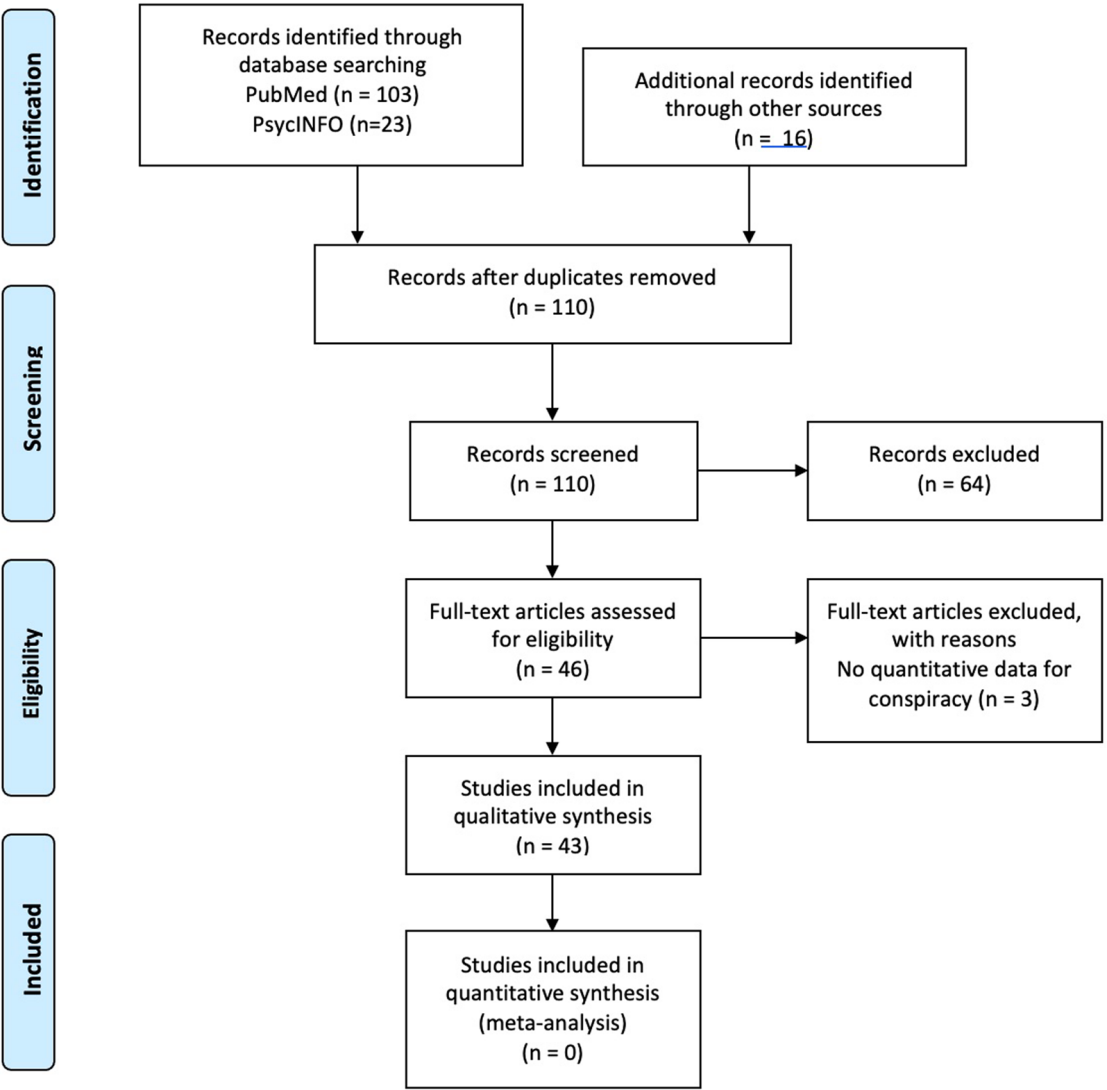
Flow Diagram of studies inclusion

Overall, 126 records were retrieved from the database searching. Additionally, 16 records were identified through other sources. Duplicates and irrelevant studies to SARS-CoV-2 were excluded; hence, a total of 110 articles were selected. After screening the full text of the articles 43 studies were eligible for inclusion (Fig. 1).

The eligible studies were published between 2020 and 2021 [8, 17–58]

### Study description and characteristics

The 43 eligible studies included a total of 14,172 posts and 61,809 participants with a median number of participants of 845 (IQR = 624  -2.057), median number of mean age of 37 years (IQR = 31- 40.2), and a median number of 58.8% of women. Eleven studies (25.6%) were conducted in the USA and seven (16.3%) in the UK. The remaining studies were conducted in various other countries as shown Tables 1 and 2. Most of the studies (88.1%) employed a cross-sectional study design using a convenience sampling method, while six studies were qualitative including analysis of tweets or posts in the social media and other sources (Table 2). No randomized studies were found. Most of the studies (58.1%) were of moderate quality. The characteristics of the included studies are summarised in Tables 1 and 2.

**Table 1:** Characteristics of quantative included studies related to COVID-19 conspiracy beliefs and theories

References	Country	Study Design	Sampling method	Sample characteristics	Measurements of Conspiracy	Conspiracy findings (Mean, SD)	Main findings of conspiracy prevalence	Study quality
Agley and Xiao [18]	USA	Cross-sectional	Convenience	N = 660, general population, Mean age = 24.8 % Female = 32.3 Ethnicity = White, Hispanic, Black or African, American,Asian and others	A list of five COVID-19 conspiracy beliefs (5G, Bill Gates, Zoonotic, Laboratory development as military weapon, Liberty restriction) using a Likert-type scale (1 = Extremely unbelievable to 7 = Extremely believable)	5G: Mean = 1.94 (SD = ± 1.72) Zoonotic: Mean = 5.56 (SD = ± 1.64) Bill Gates: Mean = 2.27 (SD = ± 1.88) Laboratory: Mean = 3.28 (SD = ± 2.00) Liberty Restriction: Mean = 2.96 (SD = ± 2.04)	Profile 1 (n = 463, 70.15%), the largest class, generally believed the scientific consensus narrative about COVID-19 and tended not to believe in other conspiracy beliefs Profile 2 (n = 54, 8.18%) considered all of the conspiracy beliefs to be highly plausible Profile 3 (n = 77, 11.67%) reported low-to-moderate believability for all of the conspiracy beliefs Profile 4 (n = 66, 10%) reported fairly high believability for most conspiracy beliefs	High
Allington et al. [29]	UK	Cross-sectional	Study 1: Convenience Study 2 and 3: Stratified random sampling	Study 1: N = 949, general population, Mean age = 36.3, Female = 68.3, Ethnicity = NR Study 2: N = 2250, general population, Mean age = 45.5, % Female = 51.3, Ethnicity = NR Study 3: N = 2254, general population, Mean age = 43.9, % Female = 49.9, Ethnicity = NR	Study 1:A list of three COVID-19 conspiracy beliefs (Laboratory development, 5G Intentional spread) Study 2: On item that coronavirus was probably created in a laboratory Study 3: A list of five COVID-19 conspiracy beliefs (Laboratory development, 5G, Intentional spread, not really exist, part of a global effort to force everyone to be vaccinated)	NR	Study 1: 24.24% believed that COVID-19 was probably created in a laboratory. 5.16% believed on 5G conspiracy theory. 9.8% believed that pandemic was planned by certain pharmaceutical corporations Study 2: 24.6% believed that coronavirus was probably created in a laboratory Study 3: 0.14% believed that COVID-19 was probably created in a laboratory. 8.13% believed on 5G conspiracy theory. 7.32% believed that coronavirus did not really exist. 13.74% believed that dying from coronavirus is being deliberately exaggerated by the authorities and 12.67% believed that pandemic is part of a global effort to force everyone to be vaccinated	Moderate
Alper [39]	Turkey	Cross-sectional	Convenience	N = 1088, general population, Mean age = 31.0 % Female = 72.6 Ethnicity = NR	A list of two COVID-19 conspiracy theories (Intentional spread or natural spread no intention) using a Likert-type scale (1 = strongly disagree, to 7 = strongly agree) and the 15-item Generic Conspiracist Beliefs Scale	Intentional spread: Mean = 2.91 (SD = ± 1.72) Generic conspiracist beliefs: Mean = 2.53 (SD = ± 0.78)	NR	High
Bertin et al. [49]	France	Cross-sectional	Convenience	Study 1:N = 409, general population, Mean age = 28.4 % Female = 73.1 Ethnicity = NR Study 2:N = 469, general population, Mean age = 26.1 % Female = 59.7 Ethnicity = NR	Study 1:A list of COVID-19 conspiracy beliefs involving a threatening foreign outgroup (e.g., China) and theories involving unspecified outgroup or members of the national ingroup (e.g., French government) Study 2: same scale as in Study 1, and added a conspiracy theory about the creation of the coronavirus by a famous French laboratory	Study 1:Outgroup COVID-19 conspiracy beliefs: Mean = 1.44 (SD = ± 0.69) Ingroup COVID-19 conspiracy beliefs: Mean = 2.47 (SD = ± 0.97) Study 2: Outgroup COVID-19 conspiracy beliefs: Mean = 1.60 (SD = ± 0.69) Ingroup COVID-19 conspiracy beliefs: Mean = 2.60 (SD = ± 0.94) Chloroquine conspiracy beliefs: Mean = 2.47 (SD = ± 0.97)	NR	High
Biddlestone et al. [54]	UK	Cross-sectional	Convenience	N = 704, general population, Mean age = 37.3 % Female = 53.4 Ethnicity = British, American, other	A list of three COVID-19 conspiracy beliefs (5G, Laboratory development, Intentional spread using a Likert-type scale (1 = strongly disagree, to 7 = strongly agree)	Overall COVID‐19 conspiracies: Mean = 1.50 (SD = ± 0.57)	NR	Moderate
Bierwiaczonek et al. [55]	USA	Longitudinal	Convenience	N = 403, general population, Mean age = 37.4 % Female = 45.9 Ethnicity = Caucasian, African American, Hispanic, Asian, Native American, other	A list of three COVID-19 conspiracy beliefs (Human made, Laboratory development as military weapon, Intentional spread using a Likert-type scale (1 = not at all, to 7 = very much)	Human made Mean = 3.14 (SD = ± 1.99) Laboratory development as military weapon Mean = 3.06 (SD = ± 1.92) Intentional spread: Mean = 2.83 (SD = ± 1.92)	NR	High
Čavojová et al. [56]	Slovakia	Cross-sectional	Convenience	N = 783, general population, Mean age = 42.0 % Female = 53.3 Ethnicity = NR	A list of various conspiracy theories (Intentional spread, Vaccination narrative,Bill Gates narrative (+ microchip) on a 5-point scale	Overall COVID‐19 conspiracies: Mean = 2.07 (SD = ± 0.87)	NR	Moderate
Chen et al. [57]	Ecuador	Cross-sectional	Convenience	N = 252, health care workers Mean age = NR, Age = > 18 years % Female = 65.5 Ethnicity = NR	A list of various conspiracy theories (Intentional spread, natural spread no intention, Laboratory accident, not sure) in a yes or no response	NR	Intentional spread: n = 61, 24.2% Natural spread: n = 52, 20.6% Laboratory accident: n = 35, 13.9% Not sure: n = 104, 41.3%	High
Duplaga [58]	Poland	Cross-sectional	Representative	N = 1002, general population, Mean age = 40.1 % Female = 50.6 Ethnicity = Polish	Items asking for opinions about three popular conspiracy theories related to COVID-19 (Genetic manipulations carried out by man, to spread panic and to achieve a political aim, as a pretext for the introduction of total surveillance of the population on a 5-point scale	NR	Genetic manipulations carried out by man: n = 459, 45.8% (both agree and decidedly agree) To spread panic and to achieve a political aim: n = 418, 42.7% (both agree and decidedly agree) As a pretext for the introduction of total surveillance of the population: n = 562, 56% (both agree and decidedly agree)	Moderate
Earnshaw et al. [19]	USA	Cross-sectional	Convenience	N = 845, general population, Mean age = 40.1 % Female = 40.9 Ethnicity = Asian, Black, Latin, White, Other	Conspiracy beliefs were measured with six items modeled after a measure of HIV conspiracy beliefs (5G,Vaccination narrative, Intentional spread as bioweapons program, no such thing as the coronavirus, government could cure coronavirus, but chooses not to for financial)	Average of conspiracy beliefs: Mean = 3.11 (SD = ± 1.80)	One or more conspiracy COVID‐19 beliefs: n = 279, 33%	High
Fountoulakis et al. [20]	Greece	Ccross-sectional	Convenience	N = 3399, general population, Mean age = 34.0 % Female = 81.1 Ethnicity = NR	A list of various conspiracy theories (5G, Laboratory development, Intentional spread) on a 5-point scale	NR	Beliefs in conspiracy theories seem widely prevalent with the more bizarre (like the relationship between COVID-19 and 5G, or the involvement of a supernatural power) enjoying lower acceptance. However, on average at least half of cases accepted at least to a moderate degree some non-bizarre conspiracy including the deliberate release of the virus as a bioweapon to deliberately create a global crisis (numbers not given)	Moderate
Freeman et al. [22]	UK	Cross-sectional	Convenience	N = 2501, general population Mean age = 46.6 % Female = 50.65 Ethnicity: White English / Welsh / Scottish / Northern Irish / British = 80.3%	Oxford Coronavirus Explanations, Attitudes, and Narratives Survey (OCEANS) coronavirus general conspiracy seven-item, a 14-item-specific coronavirus conspiracy scale on a 5-point scale	Mean total generic coronavirus conspiracy score: Mean = 3.41 (SD = ± 1.70) Μean total specific coronavirus conspiracy score: Mean = 46.1 (SD = ± 26.0) Mean total score for the four official explanations (scepticism, Conspiracy cause of the virus, The spread of the virus is a deliberate attempt, conspiracy reasons for lockdown): Mean = 12.9 (SD = ± 3.40)	50% of participants showed little evidence of conspiracy thinking, 25% showed a degree of endorsement, 15% showed a consistent pattern of endorsement, and 10% had very high levels of endorsement Conspiracy cause of the virus: 21% = the virus is a hoax 62.3% = the virus is manmade 48.9% = the virus is produced by powerful organisations (e.g., government, military) The spread of the virus is a deliberate attempt: 42.4% = to reduce the size of the global population 41.6% = by governments to gain political control 38.9% = aby a group of powerful people to make money Conspiracy reasons for lockdown: 24.2% = to stop immigration 38.9% = to control every aspect of our lives 37.7% = to impose mass surveillance 32.6% = to destabilise the nation for political gain Specific Conspiracy Beliefs: 45.4% = Coronavirus is a bioweapon developed by China to destroy the West 28.3% = The virus is a biological weapon manufactured by the United States 21% = Bill Gates has created the virus in order to reduce the world population 21.3% = Coronavirus is caused by 5 G and is a form of radiation poisoning transmitted through radio waves 28.5% = Coronavirus is being used by the government to implement a police state 24.6% = Big Pharma created coronavirus to profit from the vaccines	Moderate
Freeman et al. [21]	UK	Cross-sectional	Convenience	N = 5114, general population Mean age = 46.9 % Female = 50.35 Ethnicity: White English / Welsh / Scottish / Northern Irish / British = 79.3%	Oxford Coronavirus Explanations, Attitudes, and Narratives Survey (OCEANS) coronavirus general conspiracy seven-item, a 14-item-specific coronavirus conspiracy scale on a 5-point scale	NR	Endorsement of coronavirus conspiracy beliefs: 22.6% = the virus is a hoax 56.8% = the virus is manmade 39.5% = The spread of the virus is a deliberate attempt to reduce the size of the global population 36.5% = The spread of the virus is a deliberate attempt by governments to gain political control 35.9% = The spread of the virus is a deliberate attempt by a group of powerful people to make money 40.5% = COVID-19 is a bioweapon developed by China to destroy the West 23.6% = The virus is a biological weapon manufactured by the United States 20.7% = Bill Gates has created the virus in order to reduce the world population 24.1% = Big Pharma created COVID-19 to profit from the vaccines 33% = COVID-19 is being used by the government to implement a police state 20.1% = COVID-19 is caused by 5 G and is a form of radiation poisoning transmitted through radio waves 23.1% = The vaccine will be used to carry out mass sterilisation	Moderate
Garry [23]	UK	Cross-sectional	Representative	N = 2057, general population, Mean age = NR % Female = NR Ethnicity = English	10 statements with which respondents are asked to either agree or disagree In the ‘best practice’ condition, the response options include the following options:’’strongly disagree, disagree, slightly disagree, slightly agree, agree, strongly agree, don't know’’	NR	31.7% = Coronavirus is a bioweapon developed by China to destroy the West 13.4% = Muslims are spreading the virus as an attack on Western values 17.2% = Lockdown is a plot by environmental activists to control the rest of us 17.1% = The coronavirus vaccine will contain microchips to control the people 18.8% = The United Nations (UN) and World Health Organisation (WHO) have manufactured the virus to take global control 10.6% = Jews have created the virus to collapse the economy for financial gain	High
Georgiou et al. [24]	Multisite	Cross-sectional	Convenience	N = 640, general population, Mean age = NR, Age = > 18 years % Female = 49.5 Ethnicity = NR	A 9-item COVID-19 conspiracy scale (5G,Vaccination narrative, Intentional spread as bioweapons program) on a 7-point scale ranging from 1 = Strongly disagree to 7 = Strongly degree The beliefs in conspiracy theory inventory (BCTI) and the generalised conspiracy beliefs scale (GCBS)	Average COVID conspiracy scale: Mean = 27.0 (SD = ± 10.25), General conspiracy beliefs (GCBS): Mean = 35.10 (SD = ± 13.60), Specific conspiracy beliefs (BCTI): Mean = 50.5 (SD = ± 25.6)	NR	High
Hursh et al. [26]	USA	Cross-sectional	Convenience	N = 534, general population, Mean age = 41.9, % Female = 51.0, Ethnicity = White	Generic Conspiracist Beliefs scale (GCB)	GCB scale: Mean = 70.4 (SD = ± 21.7)	NR	High
Jolley et al. [62]	UK	Cross-sectional	Convenience	N = 534, general population, Mean age = 34.3, % Female = 49.5, Ethnicity = Britons	Belief in 5G COVID‐19 conspiracy theories was measured with five items	Belief in 5G COVID‐19: Mean = 1.93 (SD = ± 1.38)	NR	Moderate
Jovančević and Milinsevic [27]	Serbia and Latin America	Cross-sectional	Convenience	N = 412 (general population, Mean age = 31.9, % Female = 82.3, Ethnicity = 292 Serbian and 120 Latin American	One item that the virus was created in the laboratory on purpose	Belief in COVID‐19 lab conspiracy: Serbians Mean = 3.18 (SD = ± 1.24) Belief in COVID‐19 lab conspiracy: Latin Americans: Mean = 3.18 (SD = ± 1.53)	NR	Moderate
Kaparounaki [28]	Greece	Cross-sectional	Convenience	N = 1000 (general population, Mean age = 22.1, % Female = 68.0, Ethnicity = NR	Questions to assess beliefs in conspiracy theories including those pertaining to COVID-19	NR	35% believed in one or more conspiracy theories while 20% have a neutral but open approach. For covid-19, 29% believe it is a laboratory product (23% neutral), 25% that the spreading was a deliberate covert action (22% neutral) and 24% that it was developed as a bioweapon (22% neutral)	Moderate
Kim and Kim [30]	Korea	Cross-sectional	Representative	N = 3399, general population, Mean age = NR % Female = 52.1 Ethnicity = NR	A seven-item questionnaire for various beliefs in conspiracy theories	NR	17.9% of respondents supported that the government is always monitoring the public. 2.8% of respondents agreed that certain powerful nations deliberately created the coronavirus (COVID-19) to dominate the world and 8% agreed that coronavirus disease (COVID-19) was deliberately created by pharmaceutical companies to make money	High
Kowalski and Gaweda [31]	Poland	Longitudinal	ΝR	N = 110, general population, Mean age = NR % Female = 52.1 Ethnicity = NR	A 12-items questionnaire for various beliefs in conspiracy theories on a seven-point Likert scale	NR	NR	Moderate
Kowalski, et al. [32]	Poland	Cross-sectional	Proportional quota and convenience	Study 1: N = 507, general population, Mean age = 44.1 % Female = 49.9 Ethnicity = NR Study 2: N = 840, general population, Mean age = 29.9 % Female = 72.3 Ethnicity = NR	Study 1:A 14-items questionnaire for various beliefs in conspiracy theories on a seven-point Likert scale Study 1: A 12-items questionnaire for various beliefs in conspiracy theories on a seven-point Likert scale	NR	Study 1: 32.1% declared full agreement with at least one conspiracy theory (13% when government conspiracy is excluded) Study 2: 17% declared full support for at least one conspiracy theory	Moderate
Latkin et al. [33]	USA	Cross-sectional	Convenience	N = 683, general population, Mean age = 39.1 % Female = 55.5 Ethnicity = NR	One question for COVID-19 related to China purposely spread the coronavirus on a five-point Likert scale	NR	13.3% (n = 91) agreed or strongly agreed that China spread virus purposefully	High
Maftei and Holman [34]	Romania	Cross-sectional	Convenience	N = 245, general population, Mean age = NR % Female = 78.4 Ethnicity = NR	The 15-item Generic Conspiracist Beliefs Scale (GCB)	GCB: Mean = 2.93 (SD = ± 0.82)	NR	Moderate
Patsali et al. [35]	Greece	Cross-sectional	Convenience	N = 1535, university students, Mean age = 22.2 % Female = 71.9 Ethnicity = NR	A 16-items questionnaire for various beliefs in conspiracy theories on a five-point Likert scale	NR	Beliefs in conspiracy theories ranged from 20 to 68%. Less than 50% rejected six out of fifteen beliefs surveyed	Moderate
Pickles et al. [36]	Australia	Longitudinal	Representative	N = 7613, general population, Mean age = 43.3 % Female = 64.4 Ethnicity = NR	A list of various conspiracy theories (5G, Laboratory development, Intentional spread)	NR	0.6% believed that 5G networks are spreading the virus. 4.2% believed that parcels from China can spread the virus. 12.2% believed that the virus was engineered and released from a Chinese laboratory in Wuhan	Moderate
Prati [37]	Italy	Cross-sectional	Representative	N = 624, general population, Mean age = 32.3 % Female = 54.0 Ethnicity = NR	Belief about the non-natural origin of the virus based on yes; no; do not know	NR	55% (n = 343) believed that the virus is a laboratory construct and/or the product of purposeful manipulation	Moderate
Romer and Jamieson [8]	USA	Longitudinal	Representative	Wave 1: N = 1050, general population, Mean age = NR % Female = 54.4 Wave 2: N = 840, general population, Mean age = NR % Female = 55.4 Ethnicity = White, Black, Hispanic	A list of various conspiracy theories (pharmaceutical industry created the virus, laboratory development a biological weapon, the CDC exaggerating the danger to damage the Trump on a 4-point scale)	Wave 1: Average COVID‐19 conspiracy belief:Mean = 1.75 (SD = ± 0.85) Wave 2: Average COVID‐19 conspiracy belief: Mean = 1.90 (SD = ± 1.08)	14.8% believed the pharmaceutical industry created the virus, 28.3% believed the Chinese government created the virus as a bioweapon, 25.3% believed the CDC exaggerating the danger to damage the Trump	Low
Salali and Uysal [38]	UK and Turkey	Cross-sectional	Representative	UK:N = 1088 Mean age = NR % Female = NR Turkey: N = 3936 Mean age = NR % Female = NR Ethnicity = NR	Beliefs on the origin of the virus (natural/artificial/not sure)	NR	More participants in the UK believed in the natural origin of the virus (54% in Turkey, 63% in the UK, n = 5024, χ2 = 24.6, *p* < 0.001), and 18% in Turkey and 12% in the UK thought the origin to be artificial, i.e., human made	Low
Sallam et al. [40]	Jordan	Cross-sectional	Convenience	N = 1540, University students, Mean age = 22.0 % Female = 74.4 Ethnicity = most were Jordanians	On question as follows: “Do you think the COVID-19 pandemic is part of a global conspiracy theory?” (no/yes/maybe)	NR	16.4% (n = 253) of the participants stated that they believe in the role of conspiracy in the origin of the disease, and those who answered maybe represented 49.9% (n = 769) of the study population	Low
Sutton and Douglas [41]	UK	Cross-sectional	Convenience	N = 748, general population Mean age = 30.7 % Female = 67.6 Ethnicity = White, Black, Asian, mixed race	Freeman (2020) conspiracy scale on three conspiracy beliefs on a five-point (N = 251) Five-point conspiracy balanced scale on three conspiracy beliefs (N = 251) Nine-point conspiracy balanced scale on three conspiracy beliefs (N = 246)	NR	Freeman scale: 2% believed that Jews have created the virus for financial gain. 2.8% believed that Muslims are spreading the virus as an attack on Western values, and 31.9% believed that coronavirus is a bioweapon developed by China Five-point balanced scale: 0.4% believed that Jews have created the virus for financial gain. 1.6% believed that Muslims are spreading the virus as an attack on Western values, and 8.8% believed that coronavirus is a bioweapon developed by China Nine-point balanced scale: 0.8% believed that Jews have created the virus for financial gain. 0.8% believed that Muslims are spreading the virus as an attack on Western values, and 10.6% believed that coronavirus is a bioweapon developed by China	Low
Teovanović et al. [42]	Serbia	Cross-sectional	Convenience	N = 407, general population, Mean age = 34.9 % Female = 76.9 Ethnicity = NR	A 13-items questionnaire for various beliefs in conspiracy theories (e.g., 5G) on a five-point Likert scale	Average COVID‐19 conspiracy belief: Mean = 2.25 (SD = ± 0.79)	NR	Low
Sallam et al. [43]	Jordan, Kuwait and other Arab countries	Cross-sectional	Convenience	N = 3414, general population, Mean age = 31.0 % Female = 76.0 Ethnicity = Jordan, Kuwait, Saudi Arabia, Others	An 8-items questionnaire that assessed belief in conspiracy about COVID-19’s origin	NR	59.5% believed that COVID-19 is a man-made virus (n = 2031). 40% believed that COVID-19 is a man-made disease made to force everyone to get the vaccine (n = 1376), and 27.7% believed that the COVID-19 vaccine is a way to implant microchips into people to control them (n = 947)	Moderate
Sallam et al. [44]	Jordan	Cross-sectional	Convenience	N = 3150, general population, Mean age = 31.0 % Female = 76.0 Ethnicity = Jordans and non-Jordans	A list of various conspiracy theories (part of a global conspiracy, a biological warfare, 5G, spiritual divine test or trial)	NR	47.9% believed that COVID-19 is part of a global conspiracy (n = 1501). 57.0% believed that COVID-19 is part of a biological warfare (n = 1778). 21.0% believed that 5G networks have a role in COVID-19 spread (n = 641). 82.7% that COVID-19 pandemic is a divine spiritual test (n = 2595)	Moderate
Jutzi et al. [47]	USA	Cross-sectional	Convenience	Study 2 only*: N = 348, general population, Mean age = 37.7 % Female = 40.8 Ethnicity = Americans	A list of two conspiracy theories (a biological warfare, laboratory accident) on a five-point Likert scale	Average COVID‐19 conspiracy belief: Mean = 3.21 (SD = ± 0.96)	NR	Moderate
Lobato et al. [50]	USA	Cross-sectional	Representative	N = 296, general population, Mean age = 39.5 % Female = 36.2 Ethnicity = Americans	A list of various conspiracy theories (virus is a chimera Donald Trump, a biological warfare 5G) ranged from scores of 0 to 100	Average COVID‐19 conspiracy belief: Mean = 40.9 (SD = ± 28.47)	NR	Moderate
Oleksy et al. [51]	Poland	Cross-sectional	Convenience	Study 1: N = 1046, general population, Mean age = 44.1 % Female = 49.9 Ethnicity = NR Study 2: N = 1680, general population, Mean age = 26.2 % Female = 74.0 Ethnicity = NR	Studies 1 & 2: A two items questionnaire for general and government-related conspiracies	Study 1: Average COVID‐19 general conspiracy belief: Mean = 4.33 (SD = ± 1.38) Average COVID‐19 government-related conspiracies**:** Mean = 4.39 (SD = ± 1.66) Study 2: Average COVID‐19 general conspiracy belief: Mean = 3.76 (SD = ± 1.44) Average COVID‐19 government-related conspiracies: Mean = 3.48 (SD = ± 1.47)	NR	Low

^*^Study 1 did not reported data on conspiracy

*NR* Not reported

**Table 2:** Characteristics of qualitative included studies related to COVID-19 conspiracy beliefs and theories

References	Country	Study Design	Sampling method	Sample characteristics	Measurements of Conspiracy	Conspiracy findings	Main findings of conspiracy theories prevalence	Study quality
Islam et al. [17]	Multisite	Retrospective	Retrospectively collected COVID-19–related infodemic reports from online media platforms	N = 2311 reports related to COVID-19 infodemic in 25 languages from 87 countries a wide range of sources, including fact-checking agency websites, Facebook, Twitter, websites for television networks, and newspapers	Conspiracy theory defined as any statement, claim, and discussion of various theories related to the origin of SARS-CoV-2 and its malicious goals	One of the theories suggested that COVID-19 was a bioweapon and had been engineered by international agencies Multiple countries manufactured and spread the deadly coronavirus in China as part of an economic and psychological war against China	Among 2,311 reports, 182 (7.8%) were related to conspiracy theories	Moderate
Ahmed et al. [45]	Multisite	Cross-sectional	A 2-day period using the hashtag # 5Gcoronavirus	N = 6556 Twitter users	The 5G coronavirus conspiracy theory that argued that COVID-19 is caused by 5G	The majority of influential users tweeting about 5G, and COVID-19 consisted of members of the public sharing their views and opinions or news articles and videos supporting their cause	Of the 233 sample tweets, 34.8% (n = 81) of individual tweets contained views that 5G and COVID-19 were linked, 32.2% (n = 75) denounced the conspiracy theory. 65.2% (n = 152) of tweets derived from non-conspiracy theory supporters, which suggests that, although the topic attracted high volume, only a handful of users genuinely believed the conspiracy	Moderate
Havey [46]	USA	Cross-sectional	A one-week period using the Twitter API	N = 4000 tweets related to six misinformation topics about the COVID-19 pandemic	The use of hydroxychloroquine as treatment, the use of bleach as a preventative measure, Bill Gates intentionally causing the virus, the Chinese Communist Party intentionally causing the virus, and the Deep State causing the virus to ruin the economy and threaten President Trump’s re-election chances	Conservative Twitter users are driving the conversation around these misinformation topics	Tweets about hydroxychloroquine, Bill Gates, and bleach were about half neutral (44.8, 49.6, and 50%, respectively; neutral tweets are largely informative tweets that either reshare information or a link with minimal comment). Tweets about 5G were overwhelmingly neutral (71.5%), and qualitative review of this tweet corpus indicates that most of these tweets were sharing information about 5G connectivity or sharing links to information debunking the idea that 5G was “triggering the virus.” The nonneutral tweets for hydroxychloroquine, Bill Gates, bleach, and 5G were more negative than positive. Finally, tweets about the Deep State and the “Chinese Communist Party Virus” (CCPV) were predominantly negative (47.4% and 91.2%, respectively)	High
Li et al. [48]	USA	Cross-sectional	A 13-day period using the Twitter API	N = 500 tweets per day were collected from 31 December 2019 through 25 February 2020 and N = 18,000 tweets per day from 26 February to 13 March 2020	Conspiracy theory defined as any statement, claim, and discussion of various theories related to the origin of SARS-CoV-2	Tweets with conspiracy theories were more likely include group labelling and responsibility information, but less likely to mention the peril of COVID-19	4.21% of the tweets included misinformation about COVID-19 and 2.00% of tweets mentioned at least one COVID-19 conspiracy theories	Moderate
Quinn et al. [52]	Canada	Cross-sectional	A 10-day period using the hashtags #hoax and #plandemic, Instagram	N = 463 posts	Conspiracy theory defined as any statement, claim, and discussion of various theories related to the origin of SARS-CoV-2	Conspiracy theories were among the top five-most common broad themes	29.1% of the posts mentioned at least one COVID-19 conspiracy theories (n = 135/463)	Moderate
Atehortua and Patino [53]	USA	Cross-sectional	Convenience of publicly available messages from most popular digital platforms, i.e., Facebook, WhatsApp, Twitter,	N = 342 posts or messages	Conspiracy theory defined as any statement, claim, and discussion of various theories related to the origin of SARS-CoV-2	Two-thirds of the messages were suspected to disseminate conspiracy theories	27.1% of the posts mentioned at least one COVID-19 conspiracy theories (n = 93/342)	Moderate

### Conspiracy theories and beliefs—content and prevalence

**Fig. 2:**
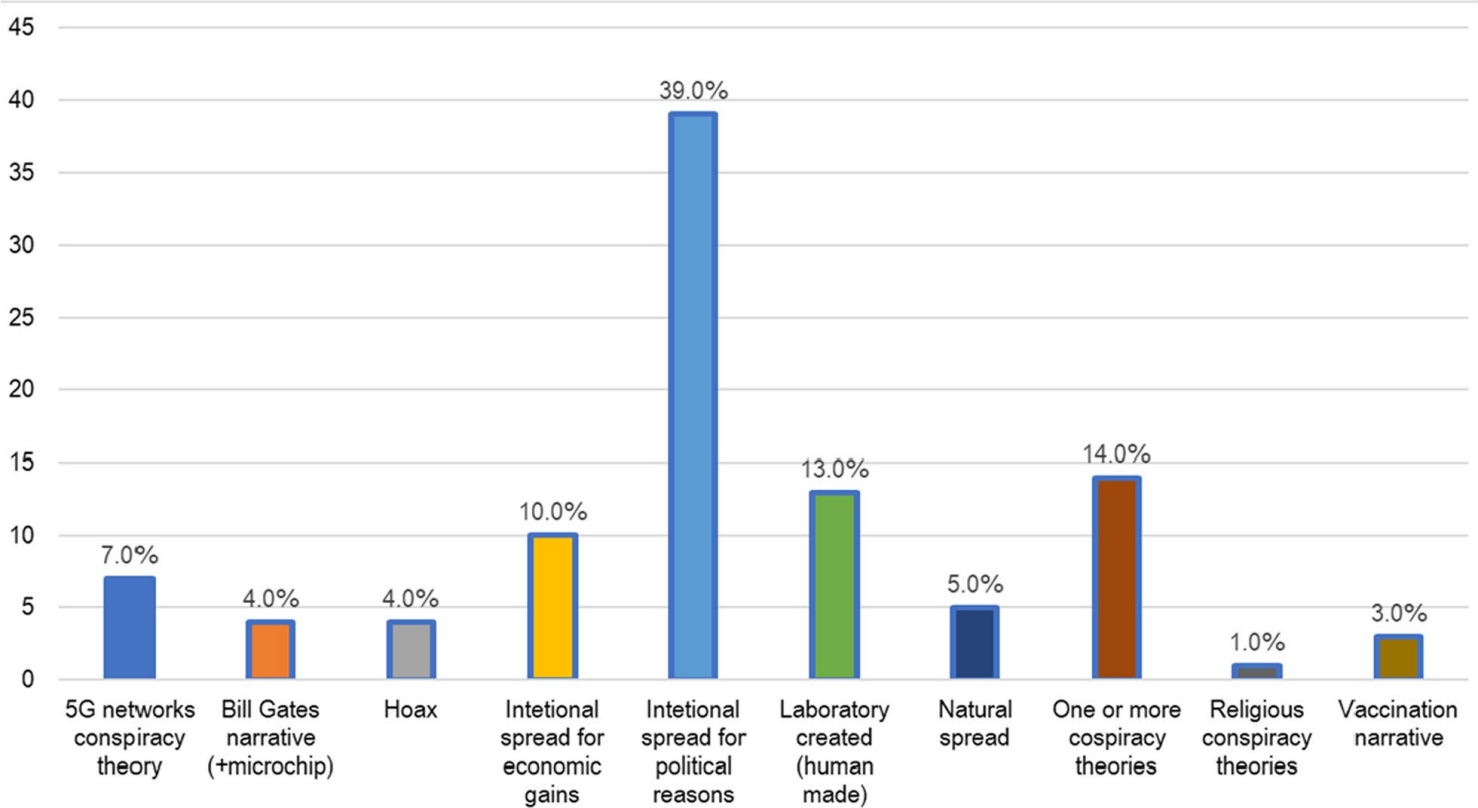
Overall percentages of various conspiracy theories

All of the studies examined various conspiracy theories, such as the 5G network theory, the theory of laboratory-created SARS-CoV-2, the theory of intentional spread of the virus, the Bill Gates/ microchip/ vaccine narrative, with the exception of one study which examined non-specific, SARS-CoV-2 related conspiracy ideation[34]. The overall percentage of participants from 28 studies (including qualitative studies) who reported agreeing with one or more conspiracy beliefs ranged from 0.4 to 82.7% [8, 17–23, 28–30, 32, 33, 35–38, 40, 41, 43–46, 48, 52, 53, 57, 58]. Because most studies provided average percentages of the different narratives calculated altogether, as well as the overlap of various conspiracy theories, it could not be determined whether certain conspiracy theories were more widespread than others. However, when we grouped them into the above-mentioned narratives/categories, only 5.0% believed in the natural origin and spread of the virus, while 39.0% believed in the intentional spread of the virus for political reasons (Fig. 2).

In regards with specific conspiracy theories, 21–34.8% of participants believed that 5G and COVID-19 were somehow linked and that 5G networks enhanced the spread of the virus [18, 44, 45]. Concerning the microchip narrative, 27.2% and 27.7% of participants in USA and Arab countries respectively believed that coronavirus vaccine will contain microchips that control people, or that COVID-19 vaccines are intended to inject microchips into recipients (and will also cause autism or infertility) [26, 43]. Theories of the virus being laboratory created were fairly widespread: only 20.6% to 29% of participants in Greece, 54% in Turkey and 63% in UK believed that SARS-CoV-2 came about naturally. At the same time, 13.9% of participants in Ecuador believed that coronavirus was created accidentally in a lab, while 24.2–58.5% of participants in Arab countries, Poland and Ecuador believed that COVID-19 was developed intentionally in a lab [43, 57, 58]. In addition, and as previously highlighted, theories of intentional spread of the virus were also quite prevalent, with 13.3% of Americans endorsing the belief that China spread the virus purposefully [33], 24% of Greeks that it was developed as a bio-weapon [28] and 57% of Jordanians that there was a biologic warfare role in the origin and spread of the virus [44]. Detailed specific conspiracy theories and their prevalence are described in Tables 1 and 2.

### Characteristics of believers in COVID-19 related conspiracy theories

There was a large heterogeneity in the factors associated with the COVID-19-related conspiracy theories, so we divided them into three categories (Table 3). Details per study are presented in Additional file 1: Table 3.

**Table 3:** Factors potentially associated with conspiracy theories and beliefs

Sociodemographic	Psychological	Other
Age Gender Ethnicity Marital Status Income Education Physical Health Status	Low tolerance of uncertainty Impulsivity Low perceived risk Lack of individual self-control Overall Conspiracy mentality/ ‘’conspiracy mindset’’ Anxiety Negative emotions Presence of depression/distress Pessimism Emotional disorders symptoms and pain Life satisfaction Anger External blame Low trust in people Persecution Boredom	Religiosity Political Orientation/Conservatism Trust in Government Scientific Reasoning Trust in Science Faith in intuition Knowledge about COVID-19 and analytic thinking Scepticism Sources and quality of information about COVID-19 Social Dominance orientation/Traditionalism Potential positive aspects of the pandemic

#### Sociodemographic characteristics potentially associated with conspiracy theories and beliefs

Several sociodemographic characteristics were associated with conspiracy theories and beliefs (see Table 3). Overall, five studies showed that conspiracy beliefs were associated with younger age [19, 22, 29, 33, 36] with effect sizes of 95% CI (− 3.22 to −  0.50), *p* = 0.007), r =  − 0.42, *p* < 0.001 and AOR = 0.97, *p* < 0.05 for the Allington, Freeman and Latkin studies respectively. One study [30] showed that age did not have a significant impact on conspiracy thinking. The majority of the studies (5 in total) showed that female gender was associated with higher belief in conspiracy theories [35, 38, 40, 43, 44], whereas only one study showed that men had stronger agreement with misinformation [36] and two studies revealed no relationship between gender and conspiracy beliefs [29, 30]. Regarding ethnicity, being white (4 studies) was associated with lower levels of conspiracy beliefs and/or increased belief in the natural origin of the virus [8, 19, 22, 38], while an Australian study found that stronger agreement with misinformation was associated with a language other than English spoken at home [36]. Furthermore, conspiracy beliefs appear to be more prevalent in those who are married (and divorced/widowed/ separated) compared to single, and to those who have children compared to those who do not [38, 44]. For example, in Sallam’s study, the belief that COVID-19 is part of a global conspiracy and the overall belief in the role of 5G networks in the spread of COVID-19 were more common among married participants compared to single participants (50.5% vs. 45.8%, *p* = 0.011; χ^2^) and (23.1% vs. 19.4% among singles, *p* = 0.017; χ^2^) respectively [44]. Another study showed that marital status had a significant association with conspiracy beliefs, but with less straightforward results [58]: more specifically, married persons were about 1.5 times more likely to believe the theory that the pandemic is used for political purposes (OR, 95% CI: 1.49, 1.02–2.17), while those who were widowed, divorced or separated were about 1.8 times more likely to believe that the pandemic is being used as a pretext for the introduction of a system of total surveillance (OR, 95% CI: 1.77, 1.08–2.91) [58].

Five studies showed that income is inversely related to conspiracy theories, i.e. higher income is related to reduced conspiracy thinking, compared to lower/middle income [8, 30, 38, 40, 44]. For example, in the Kim study [30] beliefs in conspiracy theories were high among households with incomes below 300 million won and were relatively lower in the two groups with incomes of 300 million won or more. On the other hand, one study showed no association between level of income and conspiracy thinking [58]. Furthermore, several studies (eight in total) showed an association between lower educational level and increased belief in conspiracy theories [8, 22, 24, 36, 38, 43, 44, 58]. For example, those who had a master’s degree or higher were less likely to accept the theory about the emergence of a genetically manipulated new coronavirus (OR, 95% CI: 0.5, 0.32–0.78) [58], while beliefs in COVID-19-related conspiracy theories were higher in those with a high school education compared to college degree graduates [24]. Similarly, in Salali’s study, those with postgraduate degrees had increased odds of believing in the natural origin of the virus compared to those without a graduate degree (Turkey: OR 1.63, 95% CI 1.31–2.03, *p* < 0.001, UK: OR 2.40, 95% CI 1.70–3.39, *p* < 0.001) [38]. Only one study found that education was associated with greater endorsement of conspiracy beliefs[19] and another one showed no statistical significant relationship between lower education and beliefs in conspiracy theories [30]. Interestingly, a Greek study highlighted that students of theoretical studies in particular, showed higher belief in conspiracy theories [35].

Finally, concerning physical health status, one study showed that those with better health were more likely to endorse conspiracy theories (AOR = 0.56, *p* < 0.01)[33], while another study showed that there was no correlation between health status before COVID-19 and conspiracy theories, however, there was a positive relationship with health status after COVID-19 i.e., after worsening of health status (Pearson’s r = 0.292, *p* < 0.001)[30]. Details per study are presented in Additional file 1: Table 3

#### Psychological aspects potentially associated with conspiracy theories and beliefs

As evidenced in Table 3, an array of psychological characteristics and aspects were found to predict conspiracy theories and beliefs. Details per study are presented in Additional file 1: Table 3.

For example, people who are less tolerant of uncertain situations and with higher levels of impulsivity were more likely to believe in COVID-19 conspiracy theories (r =  − 0.178, *p* < 0.001) [39]. In regards with perceived risk/perceived threat from COVID-19, three studies showed that it was inversely related with conspiracy theories [8, 36, 39] On the contrary, one study showed that beliefs in conspiracy theories were positively related to perceived risk [30]. Perceived lack of self-control had a negative effect in conspiracy theories, i.e., groups with lower perceived control had stronger beliefs in conspiracy theories [30]**.**

One study highlighted the importance of what could be called an overall conspiracy “mindset”: higher levels of coronavirus conspiracy thinking were associated with an overall conspiracy mentality, which included conspiracy beliefs about vaccines in general, climate change conspiracy theories, and an overall distrust in institutions and professions [22]. Other psychological factors that may be associated with stronger beliefs in conspiracy theories (especially the beliefs that vaccine was ready before the outbreak, biological warfare, and the role of 5G networks in the origin and spread of the virus) included higher anxiety, negative emotions, current presence of distress (OR = 2.44, 95% CI 1.20, 4.98, *p* = 0.014) [57] or depression, pessimism, emotional disorders symptoms and pain (ρ = 0.12—0.21, all *p*’s ≤ 0.001)[27, 32, 44]. However, there was inconsistency concerning the role of anxiety and stress surrounding COVID- 19. More specifically, two studies could not confirm the association between coronavirus related anxiety, self-reported stress and conspiracy beliefs [24, 32], while one study found that higher level of anxiety about COVID-19 was associated with the belief that the disease is part of a conspiracy [40] and a second study also demonstrated that people with higher anxiety had stronger beliefs in conspiracy theories [30]. In regards with depression and self-destructive behaviour, one study showed no relationship between history of depression, self-harm or suicidal attempts and any conspiracy beliefs concerning COVID-19, however, the current presence of distress or depression was significantly correlated to the belief that the vaccine was ready before the outbreak (χ^2^ = 23,088, df = 8, *p* = 0.003) and that there is a relationship to 5G (χ^2^ = 26,426, df = 8, *p* < 0.001) [20]. Interestingly, one study highlighted that health care workers who believed the virus was developed intentionally in a lab had lower life and job satisfaction than those who were unsure how the virus originated [57].

Further, another psychological factor, namely anger was related to conspiracy theories and beliefs. More specifically, beliefs in 5G/ COVID-19 conspiracy theories were significantly and positively correlated with state anger, which in turn, was associated with a greater justification of (total effect = 0.44, 95% CI[0.37, 0.52]) and willingness for (total effect = 0.19, 95% CI [0.14, 0.24]) real-life violent response to a hypothetical link between 5G networks and COVID-19 [25]. Finally, external blame, low trust in people, persecution and boredom were significantly correlated with conspiracy beliefs, as suggested by two studies [30, 32]. Details per study are presented in Additional file 1: Table 3.

#### Religion, political orientation, trust in science, sources of information and other factors potentially associated with conspiracy theories and beliefs

Four studies examined the role of religiosity and found consistent evidence that conspiracy beliefs were associated with higher religiosity (AOR = 1.12, 95% CI = 1.02–1.22) [18], (r = 0.231, *p* < 0.001) [23, 30, 39]. In addition, several studies indicated a relationship between rightist/conservative political beliefs and higher rates of conspiracy theories (r = 0.165, *p* < 0.001) [39], (AOR = 1.32, *p* < 0.01) [18, 19, 22, 30, 33, 36, 38, 46, 51]. One study showed that both ends of political spectrum (right and left) are related to increased conspiracy beliefs [23], and the same holds for those who believe that it is not worth voting in a general election [22]. Moreover, conspiracy theories appear to be linked to lower trust in government and a perception that governments and politicians are either hiding information(r = 0.28, *p* < 0.01) [24], or being dishonest about their ‘true’ intentions, in order to achieve political aims or introduce a system of total surveillance [30, 58]

With respect to scientific reasoning, analytic thinking and trust in science the results showed that these factors were inversely related to conspiracy theories (Pearson’s r = − 0.134, *p* < 0.001)[18, 19, 30, 36, 40, 42, 56]. People with greater trust in science were less likely to consider conspiracy narrative statements to be highly plausible (AOR = 0.20, 95%CI = 0.12–0.33) [18]. One study, however, found no relationship between trust in doctors and conspiracy theories [30]. Results from one study showed that belief in COVID-19 conspiracy theories was positively correlated with faith in intuition (r = 0.206, *p* < 0.001) [39]. Furthermore, reduced knowledge about COVID-19 was positively correlated with conspiracy beliefs [40]. Also, people who reported higher scepticism were less likely to believe people close to them would die from COVID-19 (AOR = 4.2, *p* < 0.01), and those who were more sceptical about COVID-19 were also more likely to believe the conspiracy theory that China purposefully spread the virus (AOR = 6.38 *p* < 0.01)[33].

Another important factor that emerged to be associated with conspiracy thinking was the source and quality of information about COVID-19. One study showed that better quality of information around COVID-19 was related to fewer conspiracy theories (Pearson’s r = 0.414, *p* < 0.001) [30]. Adding to this, several studies highlighted that use of social media as source of information on COVID-19 was related to higher levels of conspiracy thinking (95% CI (0.62–0.67, *p* < 0.001) [19, 29], (Pearson’s r = 0.134, *p* < 0.001) [8, 22, 30, 36, 43, 58]. At least three studies found that YouTube is one of the sources of information mostly associated with conspiracy beliefs [22, 29, 45]. Furthermore, one study indicated that mainstream TV news play a larger role than other news media in not legitimising COVID-related conspiracy theories [8] and similarly another study showed that use of legacy media (i.e. print media, radio broadcasting, and television) as source of information for COVID-19 was negatively associated with conspiracy theories (95% CI (0.42–0.48), *p* < 0.001) [29]. However, reliance on conservative media was positively related to endorsing conspiracies [8]. Moreover, information related to coronavirus from family and friends was associated with higher levels of conspiracy theories (95% CI (0.57–0.63), *p* < 0.001) [22, 29], while participants who endorsed conspiracies reported less trust in information coming from governmental institutions and people like Anthony Fauci [19].

Finally, one study examined the role of social dominance orientation/traditionalism and found that people with high social dominance orientation and low traditionalism were less inclined to share COVID-19 conspiracies and miscellaneous COVID-19 misinformation claims [50]. Interestingly, another study showed that people who hold COVID-19-related conspiracy beliefs were more likely to endorse positive statements about the outcomes of the pandemic [22]. These findings are summarised in Table 3 and details provided in Additional file 1: Table 3

#### Consequences and repercussions of conspiracy theories

Several studies within this systematic review reported a negative correlation between conspiracy thinking and complying with public health recommendations and public health and government measures [8, 22, 24, 29, 34, 42, 54, 55]. For example, people who reported increased belief in conspiracy theories at any wave tended to report less social distancing at the following wave [55], whereas those who endorsed the statement ‘*Coronavirus is a bioweapon developed by China to destroy the West*’ were much more likely to also not adhere (defined as less than most of the time) to ‘stay at home’ recommendations (OR 14.34, 95% CI 11.26–18.25) [22]. Greater scepticism was also strongly associated with reduced engagement in COVID-19 prevention behaviours, including confinement at home to prevent coronavirus (AOR = 0.33, *p* < 0.01) and frequently wear a mask outside (AOR = 0.44, *p* < 0.01) [33]. However, three studies showed that conspiracy beliefs were unrelated to adherence to safety guidelines [19, 31, 39]. Regarding attitudes towards the -then upcoming- vaccines there were similar findings. Results from eight studies showed that beliefs in conspiracy theories were associated with negative attitudes towards future vaccination [49] and negatively affected the intention to receive a vaccine once one became available [8, 19, 22, 37, 40, 42, 43]. Similarly one study found that believing in the natural origin of the virus significantly increased the odds of COVID-19 vaccine acceptance [38]. Details per study are presented in Additional file 1: Table 3.

## Discussion

To the best of our knowledge, the current study was one of the first to review existing evidence related to conspiracy theories about COVID-19 in the first year of the pandemic (i.e., 2020), when information about COVID-19 was more limited and still emerging, and before the systematic vaccination of the world population began. Our results suggest that the conspiracy theories were relatively common in the first year of the COVID-19 pandemic, with up to eight out of ten participants in the various surveys agreeing with at least one conspiracy theory surrounding COVID-19. This is consistent with the existing literature, which reports that conspiracy theories particularly emerge in crisis situations [59] with their prevalence increasing in times of natural disasters. Although our systematic review could not determine with certainty which conspiracy theories were the most prevalent, our results indicate that, during the first year of the pandemic, beliefs about intentional spread of the virus for political reasons were more common than others. This is in line with existing literature which showed that the majority of misinformation around a viral disease (Ebola) outbreak was of political nature[60], while previous research has indicated that groups perceived to have hostile and threatening qualities (such as politicians) can make people suspicious and thus increase conspiracy thinking [61]. Overall, it appears that in 2020, belief in theories of high implausibility (in light of logic or scientific knowledge) around COVID-19 was not a rare phenomenon.

The current study outlines specific characteristics of people who believe in conspiracy theories: those who endorse conspiracy theories are more likely to be young, female, non-white, married, physically healthy, have children, lower income and a lower level of education. Psychologically, a typical conspiracy believer tends to be more impulsive, more intolerant of uncertainty and does not perceive COVID-19 as particularly threatening. Believers of COVID-19 conspiracy theories are also more likely to have an overall conspiracy mentality (‘conspiracy mindset’) that applies to other theories and areas of life. They also exhibit increased distrust towards people, blame others, and are more likely to be depressed, angry and experience boredom. In addition, they are more likely to be religious, politically conservative and traditional, with greater skepticism and distrust in governmental and scientific institutions, while showing limited analytic thinking/reasoning and a preference for social media as their source of COVID-19 information.

Our results regarding sociodemographic factors associated with COVID-19-related conspiracy beliefs, such as young age, are in concordance with recent research; a UK-wide study from Jolley et al. [62] showed that belief in conspiracy theories flourishes in adolescents and remains constant into early adulthood. Our findings are also in agreement with existing (largely pre-COVID-19) literature, in which conspiracy theories are more prevalent in people of lower socioeconomic status [63]; previous evidence suggests that conspiracy believers tend to be less educated, have lower income, are more likely to be unemployed and members of an ethnic minority group [2, 64, 65]. One explanation could be that people with lower socioeconomic resources (and a subsequently elevated mortality risk) may experience heightened threat perceptions and out-group mistrust, and thus endorse a conspiratorial worldview [38].

In contrast to pre-COVID-19 research, which suggested that conspiracy theory believers were male and unmarried [64], our findings demonstrate that COVID-19 conspiracy theory believers were more likely to be female and married (with children). This may reflect potential gender differences in the use of social media as a source of information about COVID-19, as female social media users have been shown to exhibit less resilience to stress than their males counterparts during the COVID-19 outbreak [66]; social media use, which is nowadays more widespread than ever, has been linked to higher levels of conspiracy thinking. In addition, because women are more likely to make healthcare decisions for their children, they may also be more likely to seek out health related information [67] and are therefore exposed to conspiracy content online. Parental anxiety for the well-being of their children (given the highly transmissible nature of COVID-19, potentially putting all family members at risk) may furthermore have made married people with children more vulnerable to COVID-19-related misinformation.

It has been suggested that conspiracy theories surrounding COVID-19 can have serious, detrimental consequences in both public and individual health domains and, and it should be noted that people who believe in coronavirus conspiracy beliefs are more likely to share their opinions [22]. Our review has also shown that these conspiracy believers negatively impact pro-health behaviours such as social distancing while contributing to the undermining of preventative public health measures, like ‘stay at home’ recommendations. In particular, during the first wave/first year of the pandemic, unprecedented public health measures were taken on a global level (such as mass quarantines and full lockdowns) [68, 69]; it appears that belief in conspiracy theories may have made their implementation and effectiveness less successful. More importantly, our findings clearly showed that supporters of conspiracy theories were much more likely to refuse the -then up-coming- vaccine [70]. This is in line with pre-COVID-19 experience, as anti-vaccination conspiracy theories have played a detrimental role in negatively shaping health-related behaviours [71] and have been associated with more harmful health-related behaviours in general, such as reduced use of contraception and condoms (2). Another worrying finding is that conspiracy beliefs related to COVID-19 are more likely to be held by non-white populations, who, at the same time, are disproportionately dying from COVID-19 [72]. It is therefore not surprising that the Director-General of the World Health Organization (WHO) warned “Fake news spreads faster and more easily than this virus and is just as dangerous. We’re not just fighting an epidemic; we’re fighting an “infodemic” [73]

Actions to reduce the spread of COVID-19-related conspiracy theories are of high priority. Pre-COVID-19 promising interventions include presenting people with factual corrections and anti‐conspiracy information, and exposing disingenuous argumentation techniques [42]. During the initial stages of the COVID-19 pandemic, focus groups on critical and analytical thinking appeared to be useful in improving people’s evaluation and judgement skills [74]. However, given the unprecedented magnitude of the current “infodemic”, the solutions need to be large-scaled and convey a strong political message. Spread of misinformation needs to be combated. Collaborative initiatives between governments and the World Health Organisation, such as the ‘Stop the Spread Collaboration’, communication campaigns like ‘Reporting Misinformation’ and technological innovations (e.g. online games) that detect misinformation are absolutely vital in mythbasting” conspiracy beliefs and helping people improve their media literacy [75]. Also, given that the current review identified a number of conspiracy believers with specific characteristics, e.g., female, non-white and married people, these populations could be especially targeted for intervention.

### Limitations

Our study has certain limitations. Only studies published in English were included, which might have led to exclusion of studies published in the native language of certain countries heavily impacted by the pandemic in 2020, such as China. This also applies to the searched databases. Most of the studies included in our review used convenience sampling, which means certain groups may have been over-represented; for example, people with more social media/technology skills may have been more willing to participate in the various online surveys than for example the less technology familiar older populations. This also affects the generalisability of our results to the entire population. The presence of heterogeneity between studies in terms of tools, methods, and survey designs did not allow us to perform a quantitative synthesis of data or provide a network analysis showing the interrelationships between different variables. Also, grouping the various factors associated with conspiracy beliefs into three categories was somewhat arbitrary. Furthermore, the published studies seem to have been completed in a short time frame and therefore have not always reported significant aspects that would allow us to examine differences between groups, e.g. minorities, or to categorise the conspiracy theories according to a theoretical framework, e.g. shallow versus deep conspiracy theories [76]. Finally, the majority of the included studies were of cross-sectional design, which does not allow for establishment of casual relationships. Therefore, longitudinal studies with robust sampling methods and assessments are required.

## Conclusions

To the best of our knowledge, our study is one of the first systematic reviews examining conspiracy theories related to COVID-19 during the first year of the pandemic, when information about the virus continuously emerged. The current study found a worryingly high prevalence of COVID-19-related conspiracy beliefs during this period, including frequent beliefs about the intentional spread of the virus for political reasons, which were particularly prevalent among people with certain sociodemographic characteristics (e.g., young, non-white and female), explicit psychological traits (e.g., intolerance to uncertainty, distress and anger) and certain qualities (e.g., religiosity, conservatism and distrust in science). Our systematic review shows that conspiracy theories related to COVID-19 have negatively affected health-related behaviours and have posed a serious threat to public health and our society in general. Therefore, prompt action and cooperation between governments and health organisations are required on a global level to prevent the conspiracy theories’ adverse public health and societal consequences.

## Supplementary Information

**Additional file 1**. Supplementary Checklist 1; Supplementary Table 1: Quality assessment for quantitative studies based on the AHRQ (Agency for Healthcare Research and Quality) checklist ; Supplementary Table 2: Quality assessment for qualitative studies based on the CASP (Qualitative Research Checklist, adapted from Horntvedt et al. [15]); Supplementary Box 1: Search strategies ; Supplementary Box 2: Excluded studies with reasons; Supplementary Table 3: Factors and correlations with conspiracy theories and beliefs per study

## Data Availability

The datasets used and/or analysed during the current study are available from the corresponding author on reasonable request.
