# Supplementary material for: Summarising data and factors associated with COVID-19 related conspiracy theories in the first year of the pandemic: a systematic review and narrative synthesis
Source: BMC Psychol. 2022 Nov 1;10:244. doi: 10.1186/s40359-022-00959-6 (PMC9623972; doi:10.1186/s40359-022-00959-6)
Supplement: Supplementary file 1 — Additional file 1. Supplementary Checklist 1; Supplementary Table 1: Quality assessment for quantitative studies based on the AHRQ (Agency for Healthcare Research and Quality) checklist ; Supplementary Table 2: Quality assessment for qualitative studies based on the CASP (Qualitative Research Checklist, adapted from Horntvedt et al. [15]); Supplementary Box 1: Search strategies ; Supplementary Box 2: Excluded studies with reasons; Supplementary Table 3: Factors and correlations with conspiracy theories and beliefs per study [file 40359_2022_959_MOESM1_ESM.docx]

**Supplementary Checklist 1**

| **Section/topic** | **#** | **Checklist item** | **Reported on page #** |
| --- | --- | --- | --- |
| **TITLE** | | |  |
| Title | 1 | Identify the report as a systematic review, meta-analysis, or both. | 1 |
| **ABSTRACT** | | |  |
| Structured summary | 2 | Provide a structured summary including, as applicable: background; objectives; data sources; study eligibility criteria, participants, and interventions; study appraisal and synthesis methods; results; limitations; conclusions and implications of key findings; systematic review registration number. | 2 |
| **INTRODUCTION** | | |  |
| Rationale | 3 | Describe the rationale for the review in the context of what is already known. | 2 |
| Objectives | 4 | Provide an explicit statement of questions being addressed with reference to participants, interventions, comparisons, outcomes, and study design (PICOS). | 3 |
| **METHODS** | | |  |
| Protocol and registration | 5 | Indicate if a review protocol exists, if and where it can be accessed (e.g., Web address), and, if available, provide registration information including registration number. | 3 |
| Eligibility criteria | 6 | Specify study characteristics (e.g., PICOS, length of follow-up) and report characteristics (e.g., years considered, language, publication status) used as criteria for eligibility, giving rationale. | 3 |
| Information sources | 7 | Describe all information sources (e.g., databases with dates of coverage, contact with study authors to identify additional studies) in the search and date last searched. | 3, eBox1 |
| Search | 8 | Present full electronic search strategy for at least one database, including any limits used, such that it could be repeated. | eBox1 |
| Study selection | 9 | State the process for selecting studies (i.e., screening, eligibility, included in systematic review, and, if applicable, included in the meta-analysis). | 4-5 |
| Data collection process | 10 | Describe method of data extraction from reports (e.g., piloted forms, independently, in duplicate) and any processes for obtaining and confirming data from investigators. | 4-5 |
| Data items | 11 | List and define all variables for which data were sought (e.g., PICOS, funding sources) and any assumptions and simplifications made. | 4-5 |
| Risk of bias in individual studies | 12 | Describe methods used for assessing risk of bias of individual studies (including specification of whether this was done at the study or outcome level), and how this information is to be used in any data synthesis. | 4 |
| Summary measures | 13 | State the principal summary measures (e.g., risk ratio, difference in means). | NA |
| Synthesis of results | 14 | Describe the methods of handling data and combining results of studies, if done, including measures of consistency (e.g., I^2^) for each meta-analysis. | 4 |
| **Section/topic** | **#** | **Checklist item** | **Reported on page #** |
| Risk of bias across studies | 15 | Specify any assessment of risk of bias that may affect the cumulative evidence (e.g., publication bias, selective reporting within studies). | 6, supl matrial |
| Additional analyses | 16 | Describe methods of additional analyses (e.g., sensitivity or subgroup analyses, meta-regression), if done, indicating which were pre-specified. | NA |
| **RESULTS** | | |  |
| Study selection | 17 | Give numbers of studies screened, assessed for eligibility, and included in the review, with reasons for exclusions at each stage, ideally with a flow diagram. | Figure 1  Tables 1, 2 |
| Study characteristics | 18 | For each study, present characteristics for which data were extracted (e.g., study size, PICOS, follow-up period) and provide the citations. | Tables 1,2 |
| Risk of bias within studies | 19 | Present data on risk of bias of each study and, if available, any outcome level assessment (see item 12). | Tables 1,2 |
| Results of individual studies | 20 | For all outcomes considered (benefits or harms), present, for each study: (a) simple summary data for each intervention group (b) effect estimates and confidence intervals, ideally with a forest plot. | NA |
| Synthesis of results | 21 | Present the main results of the review. If meta-analyses are done, include for each, confidence intervals and measures of consistency. | 17 |
| Risk of bias across studies | 22 | Present results of any assessment of risk of bias across studies (see Item 15). | Tables 1,2 |
| Additional analysis | 23 | Give results of additional analyses, if done (e.g., sensitivity or subgroup analyses, meta-regression [see Item 16]). | NA |
| **DISCUSSION** | | |  |
| Summary of evidence | 24 | Summarize the main findings including the strength of evidence for each main outcome; consider their relevance to key groups (e.g., healthcare providers, users, and policy makers). | 21 |
| Limitations | 25 | Discuss limitations at study and outcome level (e.g., risk of bias), and at review-level (e.g., incomplete retrieval of identified research, reporting bias). | 23 |
| Conclusions | 26 | Provide a general interpretation of the results in the context of other evidence, and implications for future research. | 21-22 |
| **FUNDING** | | |  |
| Funding | 27 | Describe sources of funding for the systematic review and other support (e.g., supply of data); role of funders for the systematic review. | 23 |

**Supplementary Table 1** Quality assessment for quantitative studies based on the AHRQ (Agency for Healthcare Research and Quality) checklist

| Fisrt author, year | 1. Define the source of information (survey, record review) | 2. List inclusion and exclusion criteria for exposed and unexposed subjects (cases and controls) or refer to previous publications | 3. Indicate time period used for identifying patients | 4. Indicate whether or not subjects were consecutive if not population-based | 5. Indicate if evaluators of subjective components of study were masked to other aspects of the status of the participants | 6. Describe any assessments undertaken for quality assurance purposes (e.g., test/retest of primary outcome measurements) | 7. Explain any patient exclusions from analysis | 8. Describe how confounding was assessed and/or controlled | 9. If applicable, explain how missing data were handled in the analysis | 10. Summarize patient response rates and completeness of data collection | 11. Clarify what follow-up, if any, was expected and the percentage of patients for which incomplete data or follow-up was obtained | Quality Score |
| --- | --- | --- | --- | --- | --- | --- | --- | --- | --- | --- | --- | --- |
| Agley; 2021 | YES | YES | YES | YES | NO | NO | YES | YES | YES | NO | YES | 8 |
| Allington, 2020 (study 1) | YES | YES | YES | YES | NO | NO | YES | NO | NO | NO | YES | 6 |
| Allington, 2020 (study 2) | YES | YES | YES | YES | NO | NO | YES | NO | NO | NO | YES | 6 |
| Allington, 2020 (study 3) | YES | YES | YES | YES | NO | NO | YES | YES | NO | NO | YES | 7 |
| Alper, 2020 | YES | YES | NO | YES | NO | YES | YES | YES | YES | NO | YES | 8 |
| Bertin, 2020 (study 1) | YES | YES | YES | YES | NO | YES | YES | YES | YES | NO | YES | 9 |
| Bertin, 2020 (study 2) | YES | YES | YES | YES | NO | YES | YES | YES | YES | NO | YES | 9 |
| Biddlestone, 2020 | YES | NO | YES | YES | NO | YES | YES | YES | YES | NO | YES | 8 |
| Bierwiaczonek, 2020 | YES | NO | YES | YES | NO | YES | YES | YES | YES | YES | YES | 9 |
| Čavojová, 2020 | YES | NO | YES | YES | NO | NO | YES | YES | YES | NO | YES | 7 |
| Chen, 2020 | YES | NO | YES | YES | NO | NO | YES | YES | YES | YES | YES | 8 |
| Duplaga, 2020 | YES | NO | YES | YES | NO | YES | YES | YES | NO | NO | YES | 7 |
| Earnshaw, 2020 | YES | YES | YES | YES | NO | NO | YES | YES | YES | NO | YES | 8 |
| Fountoulakis, 2021 | YES | NO | YES | YES | NO | NO | YES | YES | YES | NO | YES | 7 |
| Freeman , 2020 | YES | NO | YES | YES | NO | YES | YES | YES | YES | NO | YES | 8 |
| Freeman, 2020 | YES | NO | YES | YES | NO | YES | YES | NO | YES | NO | YES | 7 |
| Garry , 2021 | YES | YES | YES | YES | NO | YES | YES | YES | YES | YES | YES | 10 |
| Georgiou , 2020 | YES | NO | YES | YES | NO | YES | YES | YES | YES | NO | YES | 8 |
| Hursh , 2020 | YES | YES | YES | YES | NO | NO | YES | YES | YES | NO | YES | 8 |
| Jolley, 2020 | YES | NO | NO | YES | NO | YES | YES | YES | YES | NO | YES | 7 |
| Jovančević, 2020 | YES | NO | YES | YES | NO | YES | YES | NO | YES | NO | YES | 7 |
| Kaparounaki, 2020 | YES | NO | YES | YES | NO | NO | YES | NO | YES | NO | YES | 6 |
| Kim, 2020 | YES | NO | YES | YES | NO | YES | YES | YES | YES | YES | YES | 9 |
| Kowalski, 2020 | YES | NO | YES | NO | NO | YES | YES | YES | YES | NO | NO | 6 |
| Kowalski, 2020 (study 1) | YES | NO | YES | YES | NO | YES | YES | YES | YES | NO | YES | 8 |
| Kowalski, 2020 (study 2) | YES | NO | YES | YES | NO | YES | YES | YES | YES | NO | YES | 8 |
| Latkin, 2021 | YES | YES | YES | YES | NO | YES | YES | YES | YES | NO | YES | 9 |
| Maftei, 2020 | Yes | Yes | Yes | Yes | N/A | No | No | Yes | No | No | N/A | 5 |
| Patsali, 2020 | Yes | Yes | Yes | Yes | N/A | No | No | Yes | No | No | N/A | 5 |
| Pickles, 2020 | Yes | Yes | Yes | Yes | N/A | No | Yes | Yes | No | No | No | 6 |
| Prati, 2020 | Yes | Yes | Yes | Yes | N/A | No | No | Yes | Yes | Yes | N/A | 7 |
| Romer, 2020 | Yes | No | Yes | Yes | N/A | No | No | Yes | Yes | Partial | Partial | 6 (half a point for partial) |
| Salali, 2020 | Yes | Yes | Yes | Yes | N/A | Unclear (more details in registered protocol but inaccessible) | No | Unclear | No | No | No | 4 |
| Sallam, 2020 | Yes | No | Yes | Yes | N/A | No | No | No | No | No | N/A | 3 |
| Sutton, 2020 | Yes | Yes | Yes | Yes | Yes (participants assigned to different conditions) | No | No | No | No | No | N/A | 5 |
| Teovanović, 2020 | Yes | Yes | Yes | Yes | N/A | No | No | Yes | No | No | N/A | 5 |
| Sallam, 2020 | Yes | Yes | Yes | Yes | N/A | No | Yes | No | Yes | Yes | N/A | 7 |
| Jutzi, 2020 | Yes | No | No | Yes | Yes (manipulation in study 2) | Yes | Yes | No | No | No | N/A | 5 |
| Lobato, 2020 | Yes | No | No | Yes | N/A | Yes | Yes | No | Yes | No | N/A | 5 |
| Oleksy, 2021 | Yes | Yes | Yes | Yes | N/A | No | No | No | No | No | N/A | 4 |

**Supplementary Table 2** Quality assessment for qualitative studies based on the CASP (Qualitative Research Checklist, adapted from Horntvedt et al., 2018)

| **CASP checklist criteria** | | | | | | | | | | | |
| --- | --- | --- | --- | --- | --- | --- | --- | --- | --- | --- | --- |
| **Selected studies** | **1** | **2** | **3** | **4** | **5** | **6** | **7** | **8** | **9** | **10** | **Assessment** |
| Islam, 2020 | Y | U | U | Y | Y | U | U | Y | Y | Y | Moderate |
| Ahmed, 2020 | Y | U | Y | Y | Y | U | Y | Y | Y | Y | Moderate |
| Havey, 2020 | Y | Y | Y | Y | Y | U | Y | Y | Y | Y | High |
| Li, 2020 | Y | U | Y | Y | Y | Y | U | Y | Y | Y | Moderate |
| Quinn, 2020 | Y | U | Y | Y | Y | Y | U | Y | Y | Y | Moderate |
| Atehortua, 2020 | Y | Y | Y | U | Y | Y | U | U | Y | Y | Moderate |

Y, yes; N, no; U, unclear/cannot tell.

CASP criteria for qualitative studies:

1. Was there a clear statement of the aims of the research?

2. Was a qualitative methodology appropriate?

3. Was the research design appropriate to address the aims of the research?

4. Was the recruitment strategy appropriate to the aims of the research?

5. Was the data collected in a way that addressed the research issue?

6. Has the relationship between researcher and participants been adequately considered?

7. Have ethical issues been considered?

8. Was the data analysis sufficiently rigorous?

9. Is there a clear statement of the findings?

10. (How valuable is the research?) *This is an open-ended question.

**Supplementary Box 1** Search strategies

| **Search query in PubMed** |
| --- |
| ((("conspiracies"[All Fields] OR "conspiracy"[All Fields]) AND ("theorie"[All Fields] OR "theories"[All Fields] OR "theory"[All Fields] OR "theory s"[All Fields])) OR (("conspiracies"[All Fields] OR "conspiracy"[All Fields]) AND ("theorie"[All Fields] OR "theories"[All Fields] OR "theory"[All Fields] OR "theory s"[All Fields])) OR (("conspiracies"[All Fields] OR "conspiracy"[All Fields]) AND ("belief s"[All Fields] OR "culture"[MeSH Terms] OR "culture"[All Fields] OR "belief"[All Fields] OR "beliefs"[All Fields])) OR (("science"[MeSH Terms] OR "science"[All Fields] OR "sciences"[All Fields] OR "science s"[All Fields] OR "sciencing"[All Fields]) AND ("denial, psychological"[MeSH Terms] OR ("denial"[All Fields] AND "psychological"[All Fields]) OR "psychological denial"[All Fields] OR "denial"[All Fields] OR "denials"[All Fields])) OR ("sceptic"[All Fields] OR "sceptical"[All Fields] OR "sceptically"[All Fields] OR "scepticism"[All Fields] OR "sceptics"[All Fields])) AND ("covid 19"[All Fields] OR "covid 19"[MeSH Terms] OR "covid 19 vaccines"[All Fields] OR "covid 19 vaccines"[MeSH Terms] OR "covid 19 serotherapy"[All Fields] OR "covid 19 serotherapy"[Supplementary Concept] OR "covid 19 nucleic acid testing"[All Fields] OR "covid 19 nucleic acid testing"[MeSH Terms] OR "covid 19 serological testing"[All Fields] OR "covid 19 serological testing"[MeSH Terms] OR "covid 19 testing"[All Fields] OR "covid 19 testing"[MeSH Terms] OR "sars cov 2"[All Fields] OR "sars cov 2"[MeSH Terms] OR "severe acute respiratory syndrome coronavirus 2"[All Fields] OR "ncov"[All Fields] OR "2019 ncov"[All Fields] OR (("coronavirus"[MeSH Terms] OR "coronavirus"[All Fields] OR "cov"[All Fields]) AND 2019/11/01:3000/12/31[Date - Publication]) OR ("sars cov 2"[MeSH Terms] OR "sars cov 2"[All Fields] OR "sars cov 2"[All Fields]) OR ("coronavirus"[MeSH Terms] OR "coronavirus"[All Fields] OR "coronaviruses"[All Fields]) OR (("corona"[All Fields] OR "coronae"[All Fields] OR "coronas"[All Fields]) AND ("virology"[MeSH Subheading] OR "virology"[All Fields] OR "viruses"[All Fields] OR "viruses"[MeSH Terms] OR "virus s"[All Fields] OR "viruse"[All Fields] OR "virus"[All Fields]))) |
| **Search query in PsycINFO** |
| TX ( conspiracy theories OR conspiracy theory OR conspiracy beliefs OR science denial OR scepticism ) AND TX ( COVID-19 OR SARS-CoV-2 OR coronavirus OR corona virus ) |

Supplementary Box 2 Excluded studies with reasons

| First author, year | Reason |
| --- | --- |
| Saribas, 2020^1^ | No quantitative data for conspiracy |
| Ahmed, 2020^2^ | No quantitative data for conspiracy |
| Naeem, 2020^3^ | No quantitative data for conspiracy |

References from excluded studies

1. Saribas D, Çetinkaya E. Pre-Service Teachers’ Analysis of Claims About COVID-19 in an Online Course. Sci Educ [Internet]. 2020 Nov 25;1–32. Available from: <http://www.ncbi.nlm.nih.gov/pubmed/33250575>
2. Ahmed W, López Seguí F, Vidal-Alaball J, Katz MS. COVID-19 and the “Film Your Hospital” Conspiracy Theory: Social Network Analysis of Twitter Data. J Med Internet Res [Internet]. 2020;22(10):e22374. Available from: <http://www.ncbi.nlm.nih.gov/pubmed/32936771>
3. Naeem S Bin, Bhatti R, Khan A. An exploration of how fake news is taking over social media and putting public health at risk. Health Info Libr J [Internet]. 2020 Jul 12; Available from: http://www.ncbi.nlm.nih.gov/pubmed/32657000

**Supplementary Table 3** Factors and correlations with conspiracy theories and beliefs per study

| **First author, year** | **Main factors associated with conspiracy theories** |
| --- | --- |
| Agley, 2021 | Controlling for race/ethnicity, gender, age, and education level, individuals with greater trust in science were less likely to be in Profile 2 (AOR=0.07, 95%CI=0.03–0.16), Profile 3 (AOR=0.20, 95%CI=0.12–0.33), and Profile 4 (AOR=0.07, 95%CI=0.03–0.15) than Profile 1. Participants with greater religious commitment were more likely to be in Profile 3 (AOR=1.12, 95% CI = 1.02–1.22) than Profile 1 |
| Allington, 2020 | **Study 1**:There was a positive relationship between holding one or more conspiracy beliefs and preference for social media over legacy media as a general source of information, U(N1 = 266, N2 = 665) = 99 987.0, p = 0.001, 95% CI (0.52–0.60).There was a very strong negative relationship between holding one or more conspiracy beliefs and following all health-protective behaviours, p < 0.001, 95% CI (0.34–0.61). The strongest effects were observed for conspiracy 2 (a connection between COVID-19 symptoms and 5G). Those holding one or more conspiracy beliefs were very slightly younger, t(604.71) = −2.69, p = 0.007, 95% CI (−3.22 to −0.50), and there was no relationship between gender and conspiracy belief, p = 0.591, 95% CI (0.80–1.50).  **Study 2:** There was a significant positive relationship between holding the conspiracy belief and frequency of checking social media for news about COVID-19, U(N1 = 468, N2 = 1221) = 343 152, p < 0.001, 95% CI (0.57–0.63).There was a significant negative relationship between holding the conspiracy belief and engagement in all health-protective behaviours, p < 0.001, 95% CI (0.39–0.66). There was also a significant negative relationship between holding the conspiracy  belief and engagement in each individual health-protective behaviour. Those holding the conspiracy belief were several years younger, t(950.90) = −6.44, p < 0.001, 95% CI (−7.54 to −4.02), while women were slightly less likely to hold the conspiracy belief, although this was not statistically significant, p = 0.214, 95% CI (0.70–1.09).  **Study 3:** There was a small but significant negative relationship between use of legacy media as a source of knowledge about COVID-19 and belief in one or more conspiracy theories, U(N1 = 884, N2 = 748) = 296 848.5, p < 0.001, 95% CI (0.42–0.48). However, the effect of television and radio considered alone [U(N1=881, N2=746)=287775.0, p<.001, 93% CI 0.41-0.46] was in most cases statistically significant, while the effect of newspapers and magazines considered alone was no t [U(N1=868, N2=747)=307658.0, P=0.063, 95% CI 0.45-0.50] .There was a strong positive relationship between use of social media platforms as sources of knowledge about COVID-19 and holding one or more conspiracy beliefs, U(N1 = 882, N2 = 748) = 424 640.0, p < 0.001, 95% CI (0.62–0.67). There was also a smaller but significant positive relationship between holding one or more conspiracy beliefs and use of friends and family as a source of information about COVID-19, U(N1 = 878, N2 = 749) = 397 473.5, p < 0.001, 95% CI (0.57–0.63).There was a strong negative relationship between holding one or more conspiracy beliefs and engagement in all health protective behaviours, p < 0.001, 95% CI (0.29–0.47).Those holding one or more conspiracy beliefs were slightly younger, t(1597.01) = −4.33, p < 0.001, 95% CI (−5.02 to−1.89). Women in the sample were slightly more likely to hold one or more conspiracy beliefs, although this was not statistically significant, p = 0.164, 95% CI (0.94–1.40). |
| Alper, 2020 | Higher faith in intuition (r = .206, p < .00) , uncertainty avoidance (r = −.178, p < .001), impulsivity (r = .062, p = .042) , perceived risk, (r = −.066, p = .029), more religious (r = .231, p < .001) more politically rightist (r = .165, p < .001), and generic conspiracist beliefs, (r = .513, p < .001) associated with a higher level of belief in COVID-19 conspiracy |
| Bertin, 2020 | Study1:Attitudes toward vaccines were negatively predicted by both “outgroup” conspiracy beliefs, β= -0.38, 95+6:6% CI[-0.50, -0.27], t = -6.64, p < 0.001, and “ingroup conspiracy beliefs, β= -0.23, 95% CI[-0.34, -0.12], t = -4.19, p < 0.001.  Study2: Outgroup conspiracy beliefs (β= -0.39, 95% CI[-0.29, -0.29], t = -7.58, p < 0.001) and ingroup conspiracy beliefs (β= -0.22, 95% CI[-0.32, -0.11], t = -3.99, p < 0.001) were negative predictors of positive attitudes toward vaccination. Outgroup conspiracy beliefs (β= -0.23, 95% CI[-0.34, -0.12], t = -4.33, p < 0.001) and ingroup conspiracy beliefs (β= -0.11, 95% CI[-0.22, -0.01], t = -1.97, p =0.05) were negative predictors of intention to get vaccinated for the disease in the future outgroup conspiracy beliefs (β= 0.29, 95% CI[0.18, 0.40], t = 5.41, p < 0.001) and ingroup conspiracy beliefs (β= 0.19, 95% CI[0.08, 0.30], t = 3.57, p < 0.001) were negative predictor of positive Attitude toward chloroquine. However, when outgroup COVID-19 conspiracy beliefs were included in the model with ingroup COVID-19 conspiracy beliefs, only outgroup COVID-19 were significantly related to all dependent variables: positive attitudes towards vaccination (β= -0.43, 95% CI[-0.47, -0.25], t = -6.46, p < 0.001); intention to get vaccinated for the disease in the future (β= -0.22, 95% CI[-0.34, -0.11], t = -3.84, p < 0.001); positive attitudes toward chloroquine (β= 0.25, 95% CI[0.14, 0.37], t = 4.35, p < 0.001) |
| Biddlestone, 2020 | Belief in COVID‐19 conspiracy theories negatively predicted social distancing intentions both directly and indirectly through feelings of powerlessness, standardized indirect effect = −0.04 (−0.05, −0.01) |
| Bierwiaczonek, 2020 | Growth curve analyses  showed that those holding more conspiracy beliefs at the beginning of the pandemic were those showing the lowest increase in social distancing. cross-lagged analyses demonstrated that people who reported more conspiracy beliefs at any wave tended to report less social distancing at the  following wave |
| Čavojová, 2020 | Scientific reasoning correlated negatively with coronavirus conspiracy beliefs (r=-.44, p<0.001). Anti-vaccination attitudes correlated positively reluctance to get a hypothetical coronavirus vaccine (r = .45, p < 0.001) |
| Chen, 2020 | Health care workers who believed that the virus was developed intentionally in a lab were more likely to experience psychological distress and anxiety disorder than those who were unsure of the origin of the virus or hose who believed the virus was created accidentally (χ21=4.24, P=.039, χ21=6.42, P=.011, and χ21=8.11, P=.004) |
| Duplaga, 2020 | Higher eHealth Literacy was associated with higher beliefs of genetic manipulations (OR, 95% CI: 1.04, 1.01–1.07), spreading panic to reach political aims (OR, 95% CI: 1.04, 1.01–1.07) and using the pandemic as a pretext for increased surveillance of the population (OR, 95% CI: 1.03, 1.01–1.06). Older people were associated with lower beliefs of ), spreading panic to reach political aims and of total surveillance (OR, 95% CI: 0.96, 0.94–0.97, and 0.98, 0.97–0.99, respectively). Married were associated with higher panic to reach political aims (OR, 95% CI: 1.49, 1.02–2.17) and widowed were associated with higher beliefs of total surveillance (OR, 95% CI: 1.77, 1.08–2.91) |
| Earnshaw, 2020 | Participants who believed conspiracies reported greater trust in COVID-19 information from social media (F(1,844) = 120.94, p < .01, Cohen’s d = 0.51) and President Trump (F(1,843) = 108.80, p < .01, Cohen’s d = 0.76) in comparison to participants who disbelieved conspiracies. Participants who believed conspiracies reported slightly less trust in information from state and local governments (F(1,843) = 19.61, p < .01, Cohen’s d = 0.32), Anthony Fauci (F(1,844) = 91.82, p < .01, Cohen’s d = 0.69), and their doctor(s) (F(1,841) = 31.04, p < .01, Cohen’s d = 0.41. participants who believed conspiracies reported that they were 3.93 times less likely to get a COVID-19 vaccine once one became available |
| Fountoulakis, 2021 | A significant relationship between the current presence of distress or depression and the belief the vaccine was ready before the outbreak (J1; p=0.003), that there is a relationship to 5G (J3; p<0.001) |
| Freeman, 2020 | Endorsement scores for all specific conspiracy items significantly positively correlated with one another, and similarly all generic conspiracy items significantly positively correlated with one another. The correlation between the specific and generic coronavirus conspiracy principal component scores was high, r = 0.84, p < 0.001. Specific conspiracy beliefs, r = −0.101, p < 0.001, and generic conspiracy beliefs, r = −0.21, p < 0.001, were both negatively associated with the total endorsement score for the official explanations. Younger participants held higher levels of both specific, r = −0.42, p < 0.001, and general, r = −0.35, p < 0.001, coronavirus conspiracy beliefs. There were lower levels of both specific, df = 407.0, t = −9.44, p < 0.001, and general, df = 426.8, t = −8.21, p < 0.001, coronavirus conspiracy concerns in those of white ethnicity compared to individuals of other ethnicities General conspiracy, r = −0.06, p = 0.002, but not specific, r = −0.01, p = 0.526, coronavirus conspiracy concerns, were associated with lower levels of education. Specific and general coronavirus conspiracy beliefs were significantly higher in those who thought it not worth voting in a general election compared with individuals who thought you should only vote if you care who wins with the latter scoring significantly higher for coronavirus conspiracy beliefs than individuals who consider it everyone's duty to vote Individuals who obtained most of their information about coronavirus from the BBC had lower levels of specific, df = 934.8, t = 11.91, p < 0.001, and general, df = 1058.2, t = 12.09, p < 0.001 coronavirus beliefs compared with those who did not . Whereas individuals who obtained most of their information about coronavirus from friends had higher levels of specific, df = 602.6, t = −5.77, p < 0.001, and general, df = 618.61, t = −6.24, p < 0.001 coronavirus beliefs compared with those who did not Similarly, individuals who obtained most of their information about coronavirus from social media had higher levels of specific, df = 1263.6, t = −12.27, p < 0.001, and general, df = 1345.61, t = −12.73, p < 0.001 coronavirus beliefs compared with those who did not Individuals who obtained most of their information about coronavirus from YouTube had higher levels of specific, df = 470.73, t = −10.39, p < 0.001, and general, df = 494.11, t = −11.06, p < 0.001 coronavirus beliefs compared with those who did not |
| Freeman, 2020 | More right-wing political views were associated with coronavirus conspiracy beliefs, B=0.093, standard error=0.007, Beta=0.17, p < 0.001, R2=0.03. A hierarchical regression showed that both the linear political item, B=−0.115, standard error=0.030, Beta=0.21, p < 0.001, and a quadratic term (the political item squared), B=0.026, standard error=0.004, Beta=0.40, p < 0.001 were significant predictors of general coronavirus conspiracy scores. |
| Garry, 2021 | Being young is by far the most important demographic predictor of conspiracy belief, mistrust, and non-adherence. Also, being low-income, urban and religious predicts being supportive of conspiracies. Being male is a strong predictor of six of the 10 adherence outcomes, and being low-income, urban and religious predict several adherence measures. |
| Georgiou, 2020 | Those who were more conspiratorial were more likely to report that the government's response was too strong, illogical and that the government had kept information from the public.COVID-19 conspiracy beliefs and specific conspiracy beliefs (BCTI scores) were lower in respondents with a college degree, but higher in those who reported only a high school education. General conspiracy belief scores (GRCS) were significantly related to COVID beliefs |
| Hursh, 2020 | Respondents reporting greater conspiracy beliefs, γ = 0.39, p < 0.001, required higher vaccine efficacy |
| Jolley, 2020 | Belief in 5G COVID‐19 conspiracy theories was positively correlated with state anger, which in turn, was associated with a greater justification of real‐life and hypothetical violence in response to an alleged link between 5G mobile technology and COVID‐19, alongside a greater intent to engage in similar behaviours in the future. These associations were strongest for those highest in paranoia |
| Jovančević, 2020 | Lower levels of general trust predict belief that COVID-19 was created on purpose for Latin-Americans. For Serbians belief that COVID-19 was created in the laboratory steams from another psychological construct not accounted for in this research |
| Kaparounaki, 2020 | NR |
| Kim, 2020 | Individuals with lower trust in the government exhibit stronger beliefs in conspiracy theories than those with higher trust (F-value = 124.413, p-value = 0.000). Conservatives had stronger beliefs in conspiracy theories than progressives (F-value = 14.635, p-value = 0.000). Higher trust in the general public was significantly associated with weaker beliefs in conspiracy theories (F-value = 31.972, p-value = 0.000). Beliefs in conspiracy theories are high among households with incomes below 300 million won and are relatively lower in the two groups with incomes of 300 million won or more (F-value = 3.368, p-value = 0.035) |
| Kowalski, 2020 | Internal motivation to self-isolation (β = −0.17, p = 0.11), GPTS-R Reference (β = −0.20, p = 0.18), coronavirus conspiracy beliefs (β = −0.15, p = 0.14) and coronavirus-related anxiety (β = 0.13, p = 0.19) were not significant |
| Kowalski, 2020 | Study 1: conspiracy beliefs and coronavirus related anxiety were significantly correlated in predicting adherence to safety guidelines  Study 2: Conspiracy beliefs and paranoid thoughts (ρ = 0.23, p < 0.001) and persecution (ρ = 0.26, p < 0.001) were significantly correlated. Conspiracy beliefs were positively correlated with SCL-27-plus subscales (ρ = 0.12 - 0.21, all p's ≤ 0.001) |
| Latkin, 2021 | Those who were more sceptical about COVID-19 were also more likely to believe the conspiracy theory that China purposefully spread the virus (aOR = 6.380 p < 0.01). Those of younger age (aOR = 0.97, p < 0.05), better health (aOR = 0.56, p < 0.01), and who identified as more politically conservative (aOR = 1.32, p < 0.01) were more likely to endorse COVID-19 Scepticism statements |
| Maftei, 2020 | Conspiracy beliefs (β = −.15) were significant predictors of the perceived risk of the SARS-CoV-2, and of the perception of the adequacy of the lockdown measures (β = −.14) . Participants’ compliance with the lockdown rules imposed emerged as negatively related to their scores on the conspiracy beliefs (β = −.13) |
| Patsali, 2020 | Females had higher beliefs in 14 out of 16 conspiracy theories. School groups differed from one another in most conspiracy theories and depressed females being the most believing subgroup, along a continuum with non-depressed males being the less believing group |
| Pickles, 2020 | Stronger agreement with misinformation was associated with younger age, male gender, lower education level, and language other than English spoken at home. Lower institutional trust, lower digital health literacy, and greater rejection of official accounts were associated with a stronger agreement with COVID-19 misinformation beliefs. |
| Prati, 2020 | Controlling for socio-demographic factors, a multinomial logistic regression model revealed that no intention to receive a vaccine was associated with lower levels of worry and institutional trust, while increased odds for responding ‘do not know’ were found among participants holding beliefs about the non-natural origin of the virus. |
| Romer, 2020 | Belief in COVID-19-related conspiracy theories was highly stable across the two periods and inversely related to the (a) perceived threat of the pandemic, (b) taking of preventive actions, (c) perceived safety of vaccination, and (d) intention to be vaccinated against COVID-19 |
| Salali, 2020 | The degree of satisfaction with government's response to the pandemic was negatively associated with the belief in the natural origin (Turkey: OR 0.77, 95% CI 0.72–0.83, p < 0.001; UK: OR 0.77, 95% CI 0.67–0.88, p < 0.001). Compared to women, men in Turkey were more likely to believe in the natural origin of the virus (Turkey: OR 1.23, 95% CI 1.07–1.41, p < 0.01; UK: OR 1.26, 95% CI 0.94–1.68, p = 0.13). Believing in the natural origin significantly increased the odds of COVID-19 vaccine acceptance. Participants without any children were 41% more likely in Turkey, and 85% in the UK to believe in the natural origin of the virus (p < 0.001). Participants with postgraduate degrees had increased odds of believing in the natural origin compared to those without a graduate degree (Turkey: OR 1.63, 95% CI 1.31–2.03, p < 0.001, UK: OR 2.40, 95% CI 1.70–3.39, p < 0.001) |
| Sallam, 2020 | Females were more inclined to have conspiracy belief compared with males (36.3% vs. 23.8%; p = 0.001; χ2 test). Those with lower income had a higher likelihood to believe that the disease is the result of a global conspiracy (74.6% answered yes or maybe among participants with an income of JOD < 500, compared with 59.0% who answered yes or maybe among participants with an income of JOD > 1000, p < 0.001; χ2 test) |
| Sutton, 2020 | NR |
| Teovanović, 2020 | Belief in COVID‐19 conspiracy theories negatively predicted adherence to COVID‐19 guidelines and positively predicted following pseudoscientific advice. Those endorsing COVID‐19 conspiracy theories may be less likely to get vaccinated when a vaccine becomes available |
| Sallam, 2021 | Belief in conspiracy regarding the origin of COVID-19 and microchip was higher among respondents who relied on social media platforms compared to those who relied on medical doctors, scientists, and scientific journals (p < 0.001). Females a higher belief in conspiracy compared to males (mean conspiracy believes: 26.3 vs. 24.1 in males; p < 0.001) |
| Sallam, 2020 | Misinformation about the origin of the pandemic (being part of a conspiracy, biologic warfare and the 5G network’s role) was also associated with higher anxiety levels. This conspiracy belief was more common among females compared to males (50.1% vs. 41.2%, p<0.001; χ2), among married participants compared to single participants (50.5% vs. 45.8%, p = 0.011; χ2) and among smokers compared to non-smokers (52.8% vs. 46.3%, p = 0.001; χ2). The gradual increase in monthly income was associated with a gradual decrease in the belief that COVID-19 is part of a global conspiracy (50.5% in those with income of <500 JOD vs. 48.2% in those with income of 500–1000 JOD vs. 43.8% among those with income of >1000 JOD, p = 0.019; χ2). For educational level, the belief in conspiracy was the highest among those with a lower educational level (50.4% among those with high school or less degree vs. 48.5% among those with an undergraduate degree, vs. 40.8% among those with postgraduate degrees, p = 0.016; χ2) |
| Jutzi, 2020 | The regression coefficient between perceived COVID-19 threat salience and conspiracy beliefs (i.e., total effect) was significant, b = 0.01, SE = 0.002, t(346) = 2.95, p = 0.003, 95% CIs [0.002, 0.01]. The indirect effect of perceived COVID-19 threat salience via behavioral inhibition levels on conspiracy beliefs was significant, b = 0.01, SE = 0.002, 95% CIs [0.004, 0.01]. Furthermore, the regression coefficient between behavioral inhibition levels and conspiracy beliefs when controlling for the variance shared by perceived COVID-19 threat salience and conspiracy beliefs was significant, b = 0.26, SE = 0.05, t(346) = 5.02, p < 0.001, 95% CIs [0.16, 0.37]. The direct effect, namely the regression coefficient between perceived COVID-19 threat salience and conspiracy beliefs, was non-significant, b = 0.001, SE = 0.003, t(346) = 0.17, p = 0.865, 95% CIs [−0.005, 0.006] |
| Lobato, 2020 | Individuals high in social dominance orientation and low in Traditionalism were less inclined to share misinformation claims regarding the severity and spread of COVID-19, but more inclined to share COVID-19 conspiracies and miscellaneous COVID-19 misinformation claims |
| Oleksy, 2021 | Study 1:While conspiracy theories about government were predicted positively by lack of personal control and negatively by sense of collective control, general conspiracy beliefs were positively predicted by both lack of personal control and sense of collective control. At the same time government related conspiracy theories (but not general conspiracy beliefs) were negatively related to protective behaviour, and general conspiracy theories (but not government related ones) were positively related to xenophobic tendencies.  Study 2: Conspiracy theories about government were predicted positively by lack of personal control and negatively by sense of collective control, and general conspiracy beliefs were positively predicted by both lack of personal control and sense of collective control. |
